# Elastase levels and activity are increased in dystrophic muscle and impair myoblast cell survival, proliferation and differentiation

**DOI:** 10.1038/srep24708

**Published:** 2016-05-31

**Authors:** N. Arecco, C. J. Clarke, F. K. Jones, D. M. Simpson, D. Mason, R. J. Beynon, A. Pisconti

**Affiliations:** 1 Department of Biochemistry, Institute of Integrative Biology, University of Liverpool, Crown Street, Liverpool L69 7ZB, UK; 2 Centre for Proteome Research, University of Liverpool, Liverpool L69 7ZB, UK; 3 Centre for Cell Imaging, University of Liverpool, Crown Street, Liverpool L69 7ZB, UK

## Abstract

In Duchenne muscular dystrophy, progressive loss of muscle tissue is accompanied by fibrosis, chronic inflammation and reduced muscle regenerative capacity. Although much is known about the development of fibrosis and chronic inflammation in muscular dystrophy, less is known about how they are mechanistically linked to loss of muscle regenerative capacity. We have developed a proteomics method to discover dystrophy-associated changes in the muscle progenitor cell niche, which identified serine proteases, and especially neutrophil elastase, as candidates. We show that elastase activity is increased in dystrophic (*mdx*^*4cv*^) muscle and impairs myoblast survival in culture. While the effect of elastase on C2C12 cell survival correlates with the kinetics of elastase-mediated degradation of the substrate to which the cells adhere, the effect of elastase on satellite cell-derived primary myoblast growth and differentiation is substrate-independent and even more dramatic than the effect on C2C12 cells, suggesting a detrimental role for elastase on myogenesis *in vivo*. Additionally, elastase impairs differentiation of both primary and C2C12 myoblasts into myotubes. Our findings evidence the importance of neutrophil-mediated inflammation in muscular dystrophy and indicate elastase-mediated regulation of myoblast behaviour as a potential mechanism underlying loss of regenerative capacity in dystrophic muscle.

Duchenne muscular dystrophy (DMD) is an X-linked genetic disorder caused by mutations in the gene that encodes the cytoskeletal protein dystrophin. Patients affected by DMD experience muscle weakness from early age and progressive muscle loss as they become older^1^,^2^ with death usually occurring in the third and fourth decade of life^3^.

The pathogenesis of DMD is complex and not fully understood. It is known that the absence of dystrophin causes the muscle fibres (myofibres) to become more sensitive to mechanical stress as dystrophin is a key component of the dystrophin-glycoprotein complex (DGC), which anchors the myofibre to the extracellular matrix (ECM). The absence of dystrophin leads to loss of DGC-mediated anchorage of the myofibres to the ECM, which in turn causes the myofibres to damage during contraction^1^,^2^,^4^,^5^. Myofibre damage and the consequent leakage of intracellular proteins attract inflammatory cells. In response to myofibre injury a first and rapid wave of neutrophil infiltration is replaced by subsequent waves of macrophages with different phenotypes until inflammation is completely resolved and newly regenerated myofibres grow and mature^6^. However, in the presence of chronic injury, such as in DMD, the various waves of inflammatory cell infiltration are not synchronized as multiple foci of injury, often very close to one another in the same muscle unit, can be triggered at any time asynchronously. This implies that the normal time course of inflammatory response is dysregulated in chronic injury leading to the presence of chronic inflammation.

Satellite cells are resident muscle progenitors defined by their anatomical position between the plasma membrane of the myofibre and the surrounding basal lamina^7^. In healthy muscle, satellite cells are mitotically quiescent, but poised to become quickly activated in response to myofibre injury. Once activated, satellite cells proliferate and differentiate then either fuse directly to the damaged myofibre to repair it or fuse to one another to generate new myofibres, called myotubes^8^. The role of myofibre regeneration, and therefore the role of satellite cells, in the pathogenesis of DMD is debated. Firstly, it is controversial whether satellite cell numbers are altered in dystrophic muscle^9^,^10^,^11^,^12^,^13^,^14^. Secondly, it is controversial whether satellite cells in dystrophic muscle are directly impaired and participate in muscular dystrophy pathogenesis by impairing myofibre regeneration or whether satellite cells simply become exhausted by the continuous cycles of degeneration/regeneration without being intrinsically impaired^12^,^15^,^16^,^17^,^18^,^19^,^20^. It is plausible that satellite cells participate in the pathogenesis of DMD, either because they are intrinsically impaired or because they become exhausted by the cycles of degeneration and regeneration. Indeed the percentage of regenerating myofibres tends to decrease progressively over time in dystrophic muscle, indicating a progressive failure in myofibre regeneration. Loss of muscle regenerative capacity is accompanied by an increase in fibrogenesis such that in DMD the muscle tissue is progressively replaced by fibrotic tissue^21^.

The mechanistic coupling of chronic inflammation and fibrogenesis has been thoroughly investigated in skeletal muscle, in a number of different injury models^6^,^21^,^22^. In contrast the coupling of chronic inflammation and myogenesis is only recently beginning to be understood and the research so far has mostly focused on the role of macrophages in the regulation of myogenesis during acute injury^23^,^24^,^25^,^26^, while the role of neutrophils in the regulation of myogenesis in response to muscle injury, and especially in chronic injury, remains poorly understood.

Here we show that Duchenne-type muscular dystrophy progression in mice is associated with a progressive accumulation of neutrophils and neutrophil-derived elastase. Additionally, we show that elastase is toxic to myogenesis leading to decreased myoblast proliferation, increased cell death and decreased myoblast differentiation, fusion and myotube growth. We show that some of these effects are partly dependent on cell adhesion to specific ECM molecules and altogether provide evidence for an additional mechanism through which chronic inflammation and fibrosis might affect DMD pathogenesis.

## Results

### The dystrophic phenotype progressively worsens over time in mdx^4cv^ mice

**Figure 1:**
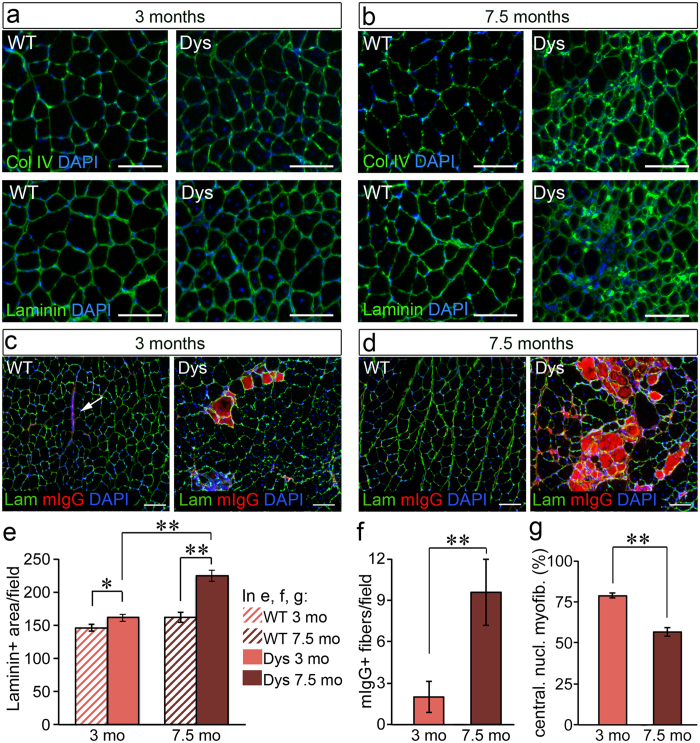
The dystrophic phenotype progressively worsens over time in mdx^4cv^ mice. (**a–d**) Gastrocnemius muscles of wild type (WT) and dystrophic (Dys, *mdx*^*4cv*^) mice at the indicated ages were dissected, cryosectioned and immunostained to detect collagen IV (**a,b**: top images, green), laminin (**a,b**: bottom images and in (**c,d**, green) and mouse immunoglobulins G (mIgG, in **c** and **d**, red). In wild type muscle mIgGs are only detected in blood vessels (arrow in **c**), while in dystrophic muscle necrotic fibres have plasma membrane leakage and uptake serum proteins such as immunoglobulins. (**e**) Quantification of laminin+ area in (**a**) and (**b**) across 10 images for each one of 3 biological replicates per age group and per genotype group (N = 30 images per data-point). (**f**) Quantification of the number of mIgG + myofibres per field as in (**c**) and (**d**) across 10 images for each one of 3 biological replicates per age group (N = 30 images per data-point). (**g**) Centrally nucleated myofibres were counted from 10 random fields imaged from 3 biological replicates per age group (N = 30 images per data-point, equal to over 3000 myofibres per data-point, scored) and plotted as average of the percentage of centrally nucleated myofibres over the total number of myofibres in the image field. Sections of all biological replicates stained for the same target were obtained at approximately the same level through the muscle length. In all graphs, error bars are S.E.M., **p < 0.01. In all images scale bars are 100 μm.

The *mdx*^*4cv*^ mouse model of DMD develops fewer dystrophin-expressing revertant myofibres than the *mdx* model^27^ and therefore shows a more severe phenotype that accumulates over time (Fig. 1a–e). We analysed the muscle phenotype of 3 month-old and 7.5 month-old *mdx*^4*cv*^ mice and observed that in the period between 3 and 7.5 months of age a switch in muscle histopathology occurs. The muscle tissue of 3 month-old *mdx*^*4cv*^ mice is characterized by a near absence of fibrosis (Fig. 1a,e), low numbers of necrotic myofibres–identified as myofibres that uptake serum proteins such as mouse immunoglobulins (Fig. 1c,f) – and high numbers of regenerating myofibres–identified as centrally-nucleated myofibres (Fig. 1a,c,g). In contrast, the muscle of 7.5 month-old mice shows signs of fibrosis–measured as abnormal accumulation of ECM proteins (Fig. 1b,e) – increased numbers of necrotic myofibres (Fig. 1d,f) and reduced numbers of regenerating myofibres (Fig. 1b,d,g). These observations suggest that after 3 months of age *mdx*^*4cv*^ mice begin to lose regenerative capacity and, concomitantly, begin to accumulate fibrotic tissue, both features becoming evident by the time the mouse reaches the age of 7.5 months. We hypothesized that loss of regenerative capacity and onset of fibrosis are mechanistically linked and that the extracellular environment established by a fibrotic and chronically inflamed tissue participates in the loss of regenerative capacity. In order to identify the mechanistic linkage between loss of regenerative capacity and onset of fibrosis, we developed a proteomics approach to characterise how the muscle extracellular environment changes as muscular dystrophy progresses.

### Detection and functional analysis of extracellular proteins in dystrophic muscle

In order to identify global changes occurring in the extracellular proteome of dystrophic muscle, we developed a method to obtain protein fractions enriched in extracellular proteins. A brief outline of the method for muscle sample preparation was previously presented^28^, however the efficiency of the method in enriching the sample preparation with extracellular proteins was not investigated. Here we provide a full description of the method and investigate its efficiency in enriching the sample preparation with extracellular proteins. Myofibre bundles from gastrocnemius muscles of wild type and dystrophic mice aged 3 and 7.5 months were obtained by microdissection of the muscle after a short incubation with collagenase to digest the epimysium and the perimysium and permit mechanical separation of myofibre groups (see Supplementary Fig. S1 and the *Methods* section for details). We then exposed these myofibre groups to trypsin to promote preferential release of extracellular proteins, which were anticipated to be more exposed to trypsin. Trypsin-released proteins were then completely digested with trypsin to generate peptides that were analysed by LC-MS/MS. The proteins were identified by MASCOT and quantified by ProgenesisQI, which was also used to calculate the p-value of differential abundance between wild type and dystrophic muscle in the two age groups. There was an excellent level of reproducibility across replicates with correlation coefficients (R^2^) between replicates of the same age and genotype on average greater than 0.98 (Supplementary Figs S2 and S3). Correlation coefficients were significantly reduced to 0.95–0.96 on average (p < 0.01) when wild type replicates were correlated to dystrophic replicates in both age groups (Supplementary Figs S2 and S3), suggesting that in both age groups, the extracellular proteome in wild type muscles was significantly different from that in dystrophic muscles.

**Figure 2:**
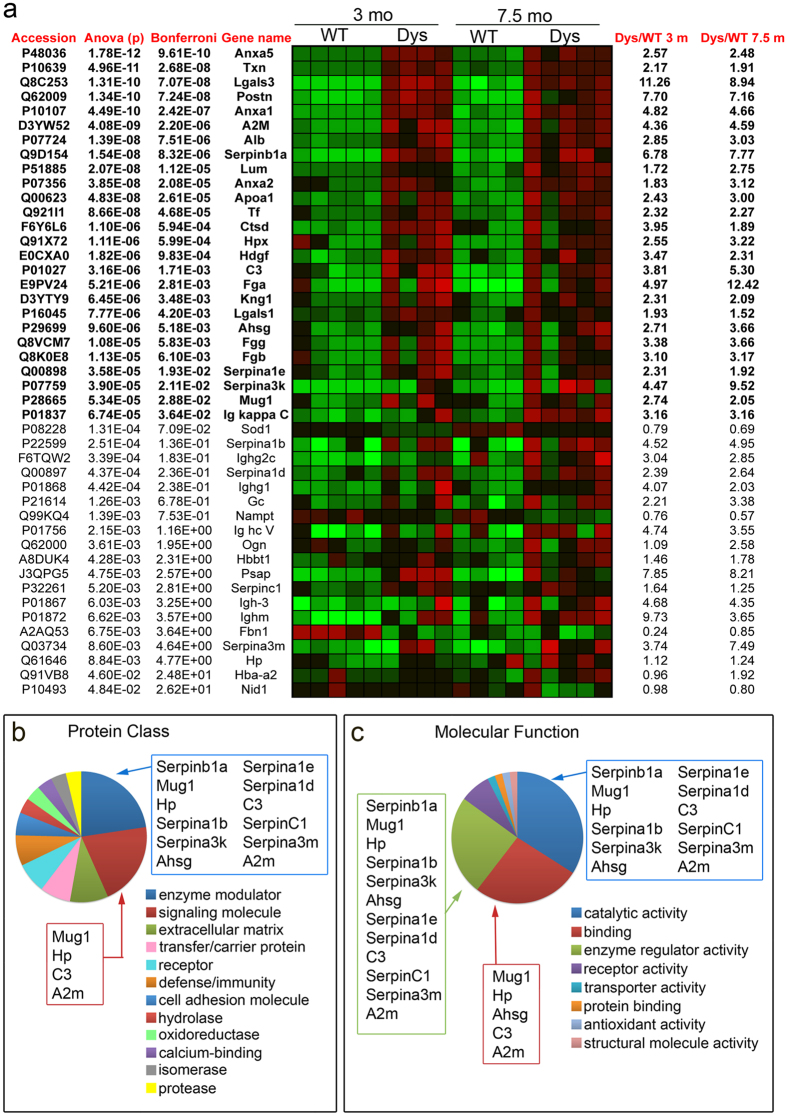
Detection of extracellular proteins in dystrophic muscle. (**a**) List of significantly different extracellular proteins organized by increasing p-value (decreasing statistical significance). Red is increased abundance, green is decreased abundance compared to the average abundance across all data-points (both ages and both genotypes) for each protein. WT = wild type, Dys = dystrophic (*mdx*^*4cv*^). (**b**) Mapping of differentially abundant extracellular proteins to *Protein class* using the functional annotation tool PANTHER (http://www.pantherdb.org/) reveals enrichment in enzyme modulators and signaling proteins, both classes including several serine protease inhibitors. (**c**) Mapping of differentially abundant extracellular proteins to *Molecular function* using the functional annotation tool PANTHER confirms the enrichment in serine protease inhibitors.

We identified a total of 568 proteins across all samples, of which 540 could be quantified through peptide ion abundance quantification (see *Methods* section for details). Using ProgenesisQI to calculate protein abundance and changes in protein abundance across replicates, we identified 322 differentially abundant proteins with a p-value <0.05 in the 3 months age group and 291 in the 7.5 months age group. When a correction for multiple testing was applied (Bonferroni correction), the number of differentially abundant proteins was 71 in the 3 months group and 38 in the 7.5 month-old group. The aim of this proteomics discovery study was to identify extracellular proteins whose abundance is significantly different in dystrophic muscle compared to wild type muscle. To understand whether our approach had succeeded in enriching the differentially abundant proteins with extracellular proteins, we mapped all proteins that were differentially abundant in either age group (q-value <0.05 by Bonferroni correction) to the Gene Ontology (GO) category *Cellular Component* using the functional analysis tool DAVID and either our list of all detected proteins (Fig. S4a) or the entire mouse genome (Fig. S4b) as background list. In both age groups *Extracellular Region* was amongst the most represented GO terms (Fig. S4a,b) in the list of differentially abundant proteins when compared to either all proteins detected (Fig. S4a) or to the entire mouse genome (Fig. S4b). Most of the extracellular proteins detected by this method were soluble proteins (Fig. 2a) that are often lost during preparation of ECM fractions. Structural proteins and proteoglycans (collagen type I, collagen type IV, collagen type VI, perlecan, lumican, fibrillin-1, nidogen-1 and periostin) were also detected but, of these, only lumican, nidogen-1, fibrillin-1 and periostin showed statistically different abundance between wild type and dystrophic muscle (Fig. 2a), suggesting that our method was more successful in detecting soluble proteins that associate with the ECM than structural ECM proteins. Thus, the method succeeded in enriching the detected proteome with extracellular and secreted proteins, although it did not completely purify the extracellular fraction. Typical cytosolic contaminants, such as the ribosomal, cytoskeletal and myofibrillar fractions, which are extremely abundant in skeletal muscle, were still represented. However, their abundance was no greater than the abundance of extracellular proteins, a direct contrast to the protein profile observed in non-enriched skeletal muscle samples, where these cytoplasmic fractions are largely dominant^29^,^30^.

Of the extracellular proteins that are significantly different at either age between wild type and dystrophic muscle (Fig. 2a), the most striking feature is that most of them (90%) are increased in dystrophic muscle compared to wild type muscle. It is not surprising that extracellular proteins are mostly increased in dystrophic muscle compared to wild type muscle since ECM accumulation is a hallmark of muscular dystrophy. Additionally, dystrophic muscle is chronically inflamed, adding to the abundance of the extracellular sub-proteome all proteins produced and secreted by immune cells.

To investigate the functional significance of the changes in abundance of extracellular proteins between wild type and dystrophic muscle, we mapped the differentially abundant proteins (proteins that changed with a p-value smaller than 0.05) to functional categories. When we mapped the differentially abundant proteins to the GO category *Protein class* using the functional analysis tool PANTHER (Fig. 2b), the most enriched protein class was *Enzyme modulator*. This protein class included several protease inhibitors, especially those belonging the family of serine protease inhibitors (Serpins) in addition to proteins involved in the acute phase response (C3 and Hp). When we mapped the differentially abundant proteins to the GO category *Molecular function* we again found that the top GO terms (*Catalytic activity, Binding* and *Enzyme regulator activity*) were highly enriched in protease inhibitors and especially Serpins (Fig. 2c).

### The Serpin family is enriched in dystrophic muscle

**Figure 3:**
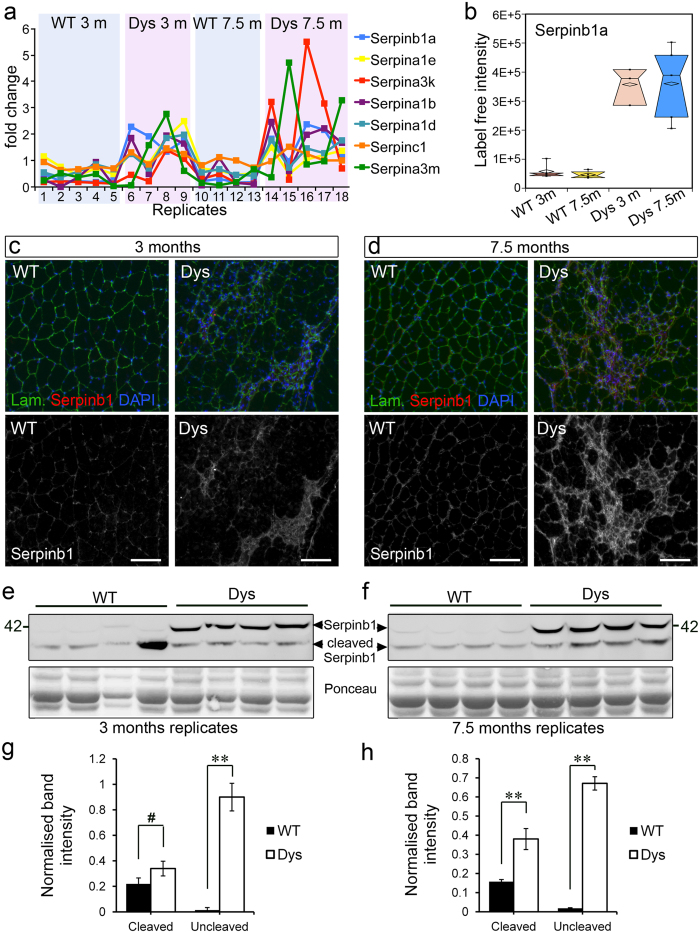
The leukocyte elastase inhibitor (Serpinb1a) is increased in dystrophic muscle from both age groups. (**a**) Seven different serine protease inhibitors (Serpins) were identified by mass spectrometry as differentially abundant in wild type and dystrophic muscle from both age groups. Five of these seven Serpins progressively accumulated with disease progression (greater increase in dystrophic over wild type muscle at 7.5 months than at 3 months of age) while two showed the opposite trend: greater increase in dystrophic over wild type muscle at 3 months than at 7.5 months of age. The most significantly different Serpin is the leukocyte elastase inhibitor Serpinb1a. (**b**) Serpinb1a is increased in dystrophic muscle at 3 and 7.5 months of age compared to age-matched wild type controls. Box plots were informed by LC-MS/MS quantification of Serpinb1a. (**c,d**) Gastrocnemius muscles of wild type (WT) and dystrophic (Dys, *mdx*^*4cv*^) mice were dissected at the indicated ages, cryosectioned and immunostained as indicated. (**e**,**f**) Western blot analysis of wild type and dystrophic (*mdx*^*4cv*^) muscle confirms increased amounts of Serpinb1a in dystrophic versus wild type muscles. (**g**) Quantification of (**e**) where the outlier wild type (lane 4) was ignored. We believe this outlier could be the consequence of an accidental muscle injury. (**h**) Quantification of (**f**). **p < 0.01; ^#^p > 0.05. Scale bars are 100 μm in (**c**,**d**).

Seven Serpins were differentially abundant in dystrophic muscle when compared to wild type muscle, covering all three main classes of mammalian serine protease activities (trypsin-, chymotrypsin- and elastase-like, Fig. 3a). The most highly significant change in Serpin abundance between wild type and dystrophic muscle was for the leukocyte elastase inhibitor Serpinb1a (Fig. 2a) that was increased over six-fold in dystrophic muscle compared to wild type muscle at 3 months and over seven-fold at 7.5 months (Figs 2a and 3b).

To validate this finding we performed immunofluorescence and Western blot analysis of wild type and dystrophic muscles using antibodies that recognize the human Serpinb1 and cross-react with the mouse analogue Serpinb1a. Immunofluorescence (Fig. 3c,d) confirmed that Serpinb1a is increased in dystrophic muscle compared to wild type muscle at both ages examined and that it tends to concentrate at sites of increased cellularity, which are the sites of focal muscle damage and inflammation (Fig. 3c,d). Serpinb1a is an endogenous inhibitor of leukocyte elastase and thus, these findings suggest that regulation of leukocyte elastase activity might be altered in dystrophic muscle. Serpinb1a inhibits neutrophil elastase and its other targets by binding to their active site in an irreversible manner, during which time Serpinb1a is also cleaved^31^. When wild type and dystrophic muscles where analysed by Western blotting for their Serpinb1a content, the uncleaved form of Serpinb1a was nearly undetectable in wild type muscles and dramatically increased in dystrophic muscle, further confirming the mass spectrometry findings (Fig. 3e–h). By contrast, the cleaved form of Serpinb1a was detected in both wild type and dystrophic muscle (Fig. 3e–h), suggesting the presence of basal levels of elastase activity in uninjured muscle. However, with the exception of one outlier sample, cleaved Serpinb1a was generally increased in dystrophic muscle compared to wild type muscle, especially at 7.5 months of age, suggesting an increase in elastase activity in the muscle of 7.5 month-old dystrophic mice (Fig. 3e–h).

### Elastase protein levels and neutrophil infiltration are increased in dystrophic muscle

**Figure 4:**
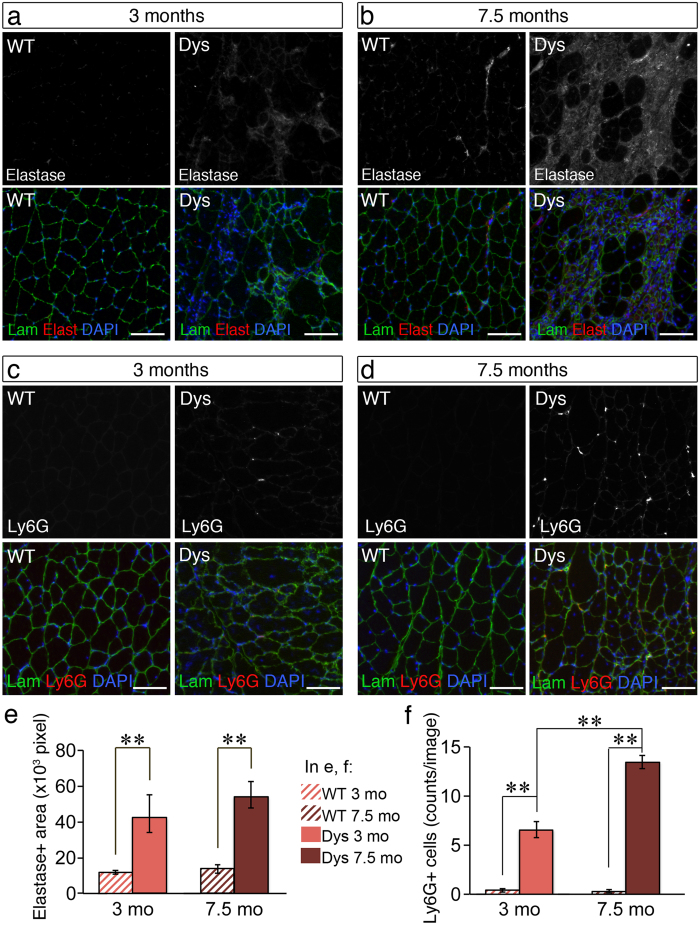
Neutrophil elastase and neutrophil numbers accumulate over time in dystrophic muscle. (**a–d**) Gastrocnemius muscles from wild type (WT) and dystrophic (Dys, *mdx*^*4cv*^) mice at 3 (**a,c**) and 7.5 (**b**,**d**) months of age were dissected, cryosectioned and immunostained to detect laminin (Lam, green), nuclei (DAPI, blue) and neutrophil elastase (**a**,**b**, Elast, red) or the murine neutrophil marker Ly6G (**c**,**d**, Ly6G, red). Both elastase levels and neutrophil numbers are increased in dystrophic muscle compared to wild type muscle and this increase is greater at 7.5 months of age (**b,d**) compared to 3 months of age (**a,c**). (**e**) Quantification of (**a,b**) where 9–12 images for each one of 3 biological replicates were analyzed and the total elastase + area per image recorded (N ≥ 27 images per data-point). (**f**) Quantification of (**c,d**) where 10–12 images for each one of 3 biological replicates were analyzed and the total number of Ly6G + cells per image recorded (N ≥ 36 images per data-point). In all graphs, error bars are S.E.M., **p < 0.01. Scale bars are 100 μm.

Leukocyte elastase is produced and released mainly by infiltrating neutrophils and thus, is expected to be increased in dystrophic muscle compared to wild type muscle due to the presence of chronic inflammation. Indeed the increase in Serpinb1a was accompanied by an increase in neutrophil elastase levels in 3 month-old dystrophic mice compared to wild type mice as measured by immunofluorescence (Fig. 4a,e), which was even more dramatic in 7.5 month-old dystrophic mice compared to age-matched wild type mice (Fig. 4b,e). Neutrophil elastase staining was observed mainly extracellularly, however it was present also inside myofibres, suggesting that it was secreted locally by phagocytic neutrophils (Fig. 4a,b). Consistent with the finding that elastase is more dramatically increased in 7.5 month-old dystrophic muscle than in 3 month-old dystrophic muscle compared to age-matched wild type controls, also neutrophil numbers, identified via immunostaining for the murine neutrophil marker Ly6G, were more numerous in 7.5 month-old dystrophic muscle compared to 3 month-old dystrophic muscle (Fig. 4c,d,f).

The data shown so far indicate a sustained presence of neutrophil elastase and its endogenous inhibitor Serpinb1a in dystrophic muscle that is dramatically reduced in wild type muscle. We postulate that this increase in elastase and Serpinb1a in dystrophic muscle is caused by a continuous influx of neutrophil in the chronically injured muscle, which participate in the establishment of chronic inflammation. This contrasts with the scenario observed during acute injury when the wave of neutrophil infiltration into injured muscle dissipates within 2–3 days^6^. To test whether the levels of elastase and Serpinb1a increased in wild type, acutely injured muscle following the same temporal pattern as neutrophil infiltration, we injected BaCl_2_ into the tibialis anterior (TA) of wild type mice and harvested both injured and contralateral uninjured TAs at 2, 4, 7 and 14 days following injury. We then analysed the levels of Serpinb1a and elastase by Western blotting (Fig. S5). As expected, both elastase and uncleaved Serpinb1a were undetectable in uninjured muscle, while cleaved Serpinb1a was present, though at low levels (Fig. S5), confirming the presence of basal levels of Serpinb1a-sensitive protease activity in uninjured muscle. Elastase levels were dramatically increased at day 2 following injury, accompanied by an increase in the levels of Serpinb1a, which was mostly in its cleaved form. As regeneration progressed, elastase levels rapidly decreased, becoming undetectable as early as 4 days following injury (Fig S5a). Over the same period, the levels of cleaved Serpinb1a also rapidly decreased (Fig. S5a,c), while the levels of total Serpinb1a decreased more slowly, effectively producing a second peak at 7 days post-injury (Fig. S5d) coincident with the peak of M1 macrophage infiltration (Figs S5b and 6). Thus, a rapid increase in elastase and Serpinb1a normally occurs during injury-induced muscle regeneration but both usually resolve within a few days after injury (Fig. S5), as the wave of neutrophil infiltration dissipates. In contrast, chronically injured muscle shows a steady-state increase in both elastase and Serpinb1a levels which tend to further accumulate over time (Figs 3 and 4).

### Elastase activity is increased in dystrophic muscle

**Figure 5:**
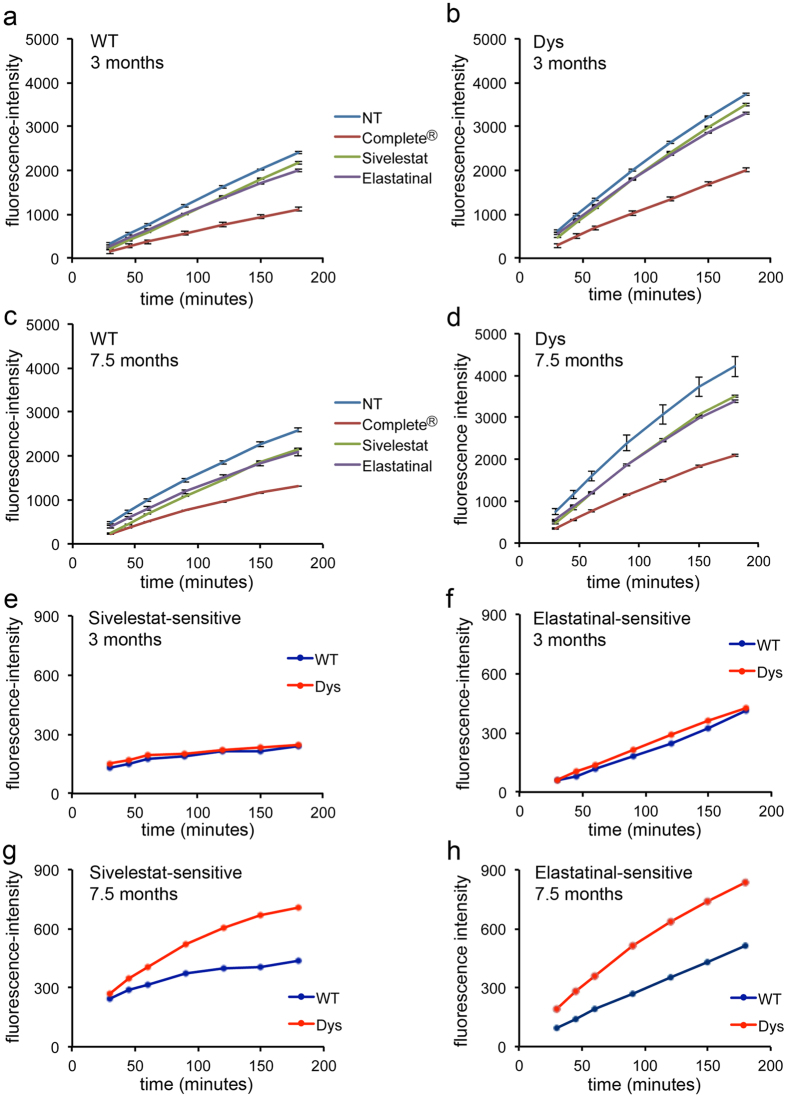
Elastase activity is increased in 7.5 month-old dystrophic muscle compared to age-matched wild type muscle. (**a**–**d**) Protease activity against the elastase substrate Suc-Ala-Ala-Ala-AMC was measured in the homogenate of wild type and dystrophic quadriceps muscles, in the presence/absence of various protease inhibitors, for each age and genotype group: 3 month old (**a,b**) and 7.5 month old (**c,d**), wild type (WT, **a**,**c**) and dystrophic (Dys, **b**,**d**) muscle. Kinetics of the accumulation of the reaction product over time (calculated as fluorescence emitted by each sample–fluorescence emitted by a blank containing only buffer and fluorogenic peptide) was plotted as the average of 3 technical replicates from 4 pooled biological replicates. Error bars are S.E.M. (**e**–**h**) The fraction of enzymatic activity against Suc-Ala-Ala-Ala-AMC that was sensitive to the elastase inhibitors Sivelestat (**e,g**) and elastatinal (**f,h**) was extracted from the averaged data shown in (**a–d**) and plotted as amount of reaction product over time: no difference in Sivelestat-sensitive or elastatinal-sensitive elastase activity is observed at 3 months of age between wild type and dystrophic muscle (**e,f**) while an increase in both Sivelestat-sensitive and elastatinal-sensitive elastase activity is observed at 7.5 months of age in dystrophic versus wild type muscle (**g,h**).

The protein levels of both elastase and the elastase inhibitor Serpinb1a are constantly increased in dystrophic muscle. To understand whether the increase in endogenous elastase inhibitor is able to completely neutralize the increase in elastase thus protecting the tissue from it, we measured elastase activity in muscle extracts from wild type and dystrophic mice. We incubated muscle homogenates with a fluorogenic elastase substrate, Suc-Ala-Ala-Ala-AMC, which produces a fluorescent signal when cleaved by elastase^32^ (Fig. 5). When a cocktail of protease inhibitors was added to the homogenate prior to incubation with the fluorogenic substrate, the activity levels were reduced by approximately 50% in both wild type and dystrophic samples (Fig. 5a–d). In contrast, the selective neutrophil elastase inhibitors Sivelestat and elastatinal were less effective at inhibiting Suc-Ala-Ala-Ala-AMC cleavage by muscle extracts especially at 3 months of age (Fig. 5a–d). Since both Sivelestat and elastatinal were used at high concentration (100 μM), these results suggest that only a fraction of the enzymatic activity measured by Suc-Ala-Ala-Ala-AMC cleavage in muscle extracts is due to elastase activity. To calculate the amount of activity that is truly attributable to elastase, we extracted the Sivelestat-sensitive (Fig. 5e,g) and elastatinal-sensitive (Fig. 5f,h) fractions from the total amount of enzymatic activity measured in the kinetic assays. When only the truly elastase activity was considered, muscles from 3 month old mice, both wild type and dystrophic, showed low levels of activity (Fig. 5e,f). In contrast, muscles from 7.5 month-old mice showed overall higher levels of elastase activity than muscles from 3 month-old mice, which were higher in dystrophic muscles compared to wild type muscles (Fig. 5g,h). These results show that elastase activity in dystrophic muscle progressively increases with disease progression, which is consistent with increasing levels of elastase proteins and numbers of neutrophil infiltrates as the disease progresses (Fig. 4).

### Elastase impairs myoblast cell survival and proliferation

**Figure 6:**
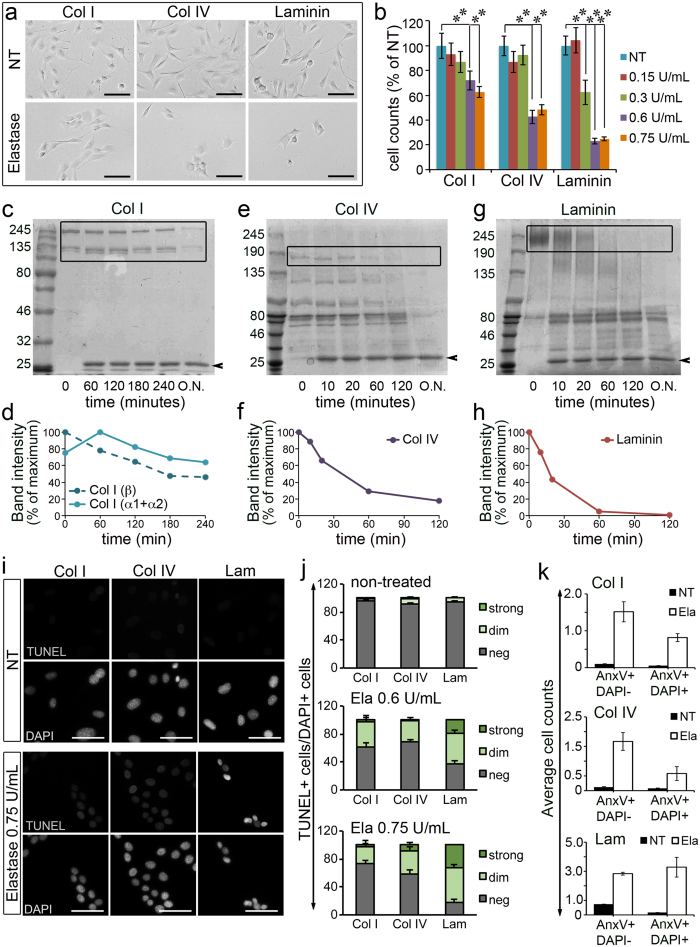
Elastase activity is toxic to cultured myoblasts in a substrate-dependent manner. (**a**) C2C12 myoblasts were treated with 0.6 U/mL elastase (NT = non-treated) for 2 days prior to fixation and imaging. (**b**) Quantification of 10–15 technical replicates for 3 biological replicates (N > 30 images per data-point) treated as in (**a**) with increasing concentrations of elastase as indicated. (**c–h**) 75 μg of collagen I from rat tail (**c,d**), collagen IV from human placenta (**e,f**) and laminin from Engelbreth-Holm-Swarm murine sarcoma (**g,h**) were incubated with 20 μg of purified elastase and sampled at the indicated time points, boiled with Laemli sample buffer then run on an 8% SDS-PAGE (**c**) or a two-phase 10–6% SDS-PAGE (**e,g**). From (**c**) the bands at ~120 kDa corresponding to Col I α1 and α2 chains and the band at ~200 kDa corresponding to the double chain β resulting from cross-linked single chains were quantified and plotted in (**d**). From (**e**) the band at ~160 kDa corresponding to Col IV (any α chain) was quantified and plotted in (**f**). From (**g**) the band at ~250 kDa corresponding to laminin was quantified and plotted in (**h**). Lower molecular weight bands, possibly corresponding to impurities, were ignored. Quantified bands are highlighted by a box in (**c,e,g**). The arrowhead pointing at a ~26 kDa band indicates elastase. (**i**,**j**) TUNEL assay of C2C12 cells treated with elastase (0.75 U/mL) for 24 hours. Representative images from one of three independent experiments are shown in (i) and quantification of 10 image fields per experiment, across three independent experiments (N = 30), in (**j**). The p-values for elastase-treated samples versus non-treated samples were all significant except for “strong staining” in Col I and Col IV, 0.6 U/mL elastase. (**k**) DAPI/Annexin-V staining of cells treated with elastase 0.75 U/mL (white bars) or untreated (black bars) 16 hours after treating. The gating scheme is shown in Fig. S7a. p-values could not be accurately calculated due to the relatively small sample size (N = 3 independent experiments). Error bars are S.E.M., **p < 0.01. Scale bars are 100 μm in (**a**) and 50 μm in (**i**).

Our data indicate that an important change occurs between 3 and 7.5 months in the muscle of *mdx*^*4cv*^ dystrophic mice: an increase in the chronic presence of neutrophils and consequently an increase in elastase protein levels and activity. Since these changes are accompanied by a reduction in regenerative potential, we asked whether elastase activity affects muscle progenitor cells. Elastase can degrade collagens and laminins, which are the main ECM components to which muscle progenitor cells adhere, both when they are in the satellite cell niche and when they migrate into the interstitial space between myofibres. The satellite cell niche is rich in laminin and collagen type IV while the interstitial space between myofibres is rich in collagen type I. Thus, activated and proliferating satellite cells, called myoblasts, are in contact with laminin, collagen type IV and collagen type I, which are all elastase targets. When we seeded C2C12 myoblasts on laminin, collagen type IV or collagen type I and treated them with increasing concentrations of elastase we observed that elastase treatment led to reduced myoblast numbers in a dose-dependent and substrate-dependent manner (Fig. 6a,b). Myoblasts adhering to laminin and collagen type IV were more sensitive to elastase-mediated toxicity than myoblasts adhering to collagen type I (Fig. 6a,b). Moreover, we observed that treatment with elastase induced cell aggregation, especially in cultures seeded on collagen type I and collagen type IV (Fig. 6a).

The substrate-dependence of the effect of elastase on myoblast numbers could be explained by different elastase selectivity for laminin versus collagens. To test this hypothesis we measured the kinetics of elastase-mediated degradation of laminin, collagen type I and collagen type IV. Indeed, we found that elastase cleaves laminin more rapidly than it cleaves collagen type I (Fig. 6c,d,g,h), while the kinetics of collagen type IV cleavage appears intermediate between that of collagen type I and that of laminin (Fig. 6e,f).

Cell adhesion provides survival signals and loss of cell adhesion can induce cell death in myoblasts both *in vivo* and *in vitro*^33^. To test whether reduced cell numbers consequent to elastase treatment was due to cell death we performed a TUNEL assay on cells cultured on various substrates in the presence/absence of elastase. We found that elastase induces myoblast cell death in a substrate-dependent manner: myoblasts seeded on laminin showed a strong positive TUNEL signal at the two concentrations of elastase examined (0.6 U/mL and 0.75 U/mL), while myoblasts seeded on collagen type I or type IV were mostly negative or only modestly positive to TUNEL (Fig. 6i,j).

Elastase could induce cell death in myoblasts seeded on laminin because laminin degradation might lead to either loss of adhesion or generation of toxic laminin fragments. To test whether elastase-dependent cell death was induced via generation of toxic fragments, we incubated myoblasts in suspension for 24 hours in the presence/absence of elastase and in the presence/absence of laminin and then re-plated them to test their viability. No significant differences in cell numbers were observed between cells treated with either elastase alone or with elastase and laminin (Fig. S6), suggesting that elastase-induced cell death is dependent on cell adhesion.

If elastase induces cell death via loss of cell adhesion, then we would expect large numbers of the cells detached upon elastase treatment to be dead. To test this, we measured plasma membrane permeability and the externalization of phosphatidylserine (via DAPI/Annexin-V staining, Fig. S7a) in the detached cell populations obtained upon treatment of adherent C2C12 cultures with elastase. As expected, elastase treatment increased cell detachment (Fig. S7b) and, consistent with the finding that reduction of cell numbers was more striking in laminin-seeded cultures, the latter yielded greater numbers of detached cells upon treatment with elastase when compared to cultures seeded on collagen type I or collagen type IV (Fig. S7b). Of these detached cells, 25–50%, depending on the substrate the cells were seeded on, were either early apoptotic (Annexin-V+/DAPI-) or late apoptotic (Annexin-V+/DAPI+) (Fig. 6k). Although similar percentages of dead cells were found in non-treated cultures, corresponding to a small number of cells that spontaneously detached and died, the fact that elastase-treated cultures contained 10–20 fold more detached cells than non-treated cultures, led to greater numbers of either early or late apoptotic cells in the detached populations of elastase-treated cultures compared to non-treated cultures (Fig. 6k). These results further support the notion that elastase-induced loss of adhesion is associated with significant levels of cell death.

### Elastase reduces myoblast proliferation, MyoD1 expression, myoblast fusion and myotube growth in a substrate-independent manner

**Figure 7:**
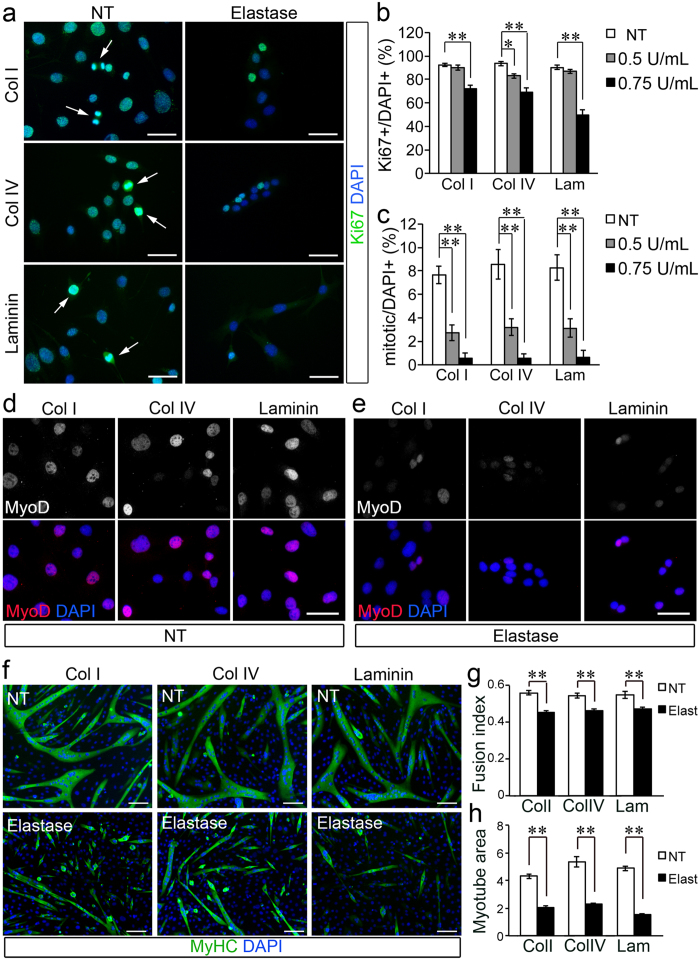
Elastase impairs myoblast proliferation, MyoD1 expression, myoblast fusion and myotube growth in a substrate-independent manner. (**a**) C2C12 myoblasts were cultured in growth medium in the presence/absence of 0.75 U/mL elastase for 24 hours then fixed and immunostained as indicated (NT = non-treated). Arrowheads show examples of mitotic cells identified by strong Ki67 staining and condensed chromatin. (**b,c**) Quantification of (**a**) where myoblasts were cultured in the presence/absence of either 0.5 or 0.75 U/mL elastase. Plots in (**b,c**) are averages of 10 technical replicates for each one of 3 biological replicates (n = 30 images per data-point). Error bars are S.E.M., *p < 0.05, **p < 0.01. Scale bars: 30 μm. (**d,e**) The levels of MyoD1 are decreased in myoblasts treated with elastase. C2C12 myoblasts were cultured in growth medium for 1 day then for an additional 1 day in the presence/absence of 0.6 U/mL elastase as indicated (NT = non-treated) prior to fixation and immunostaining to detect MyoD1 and nuclei (DAPI) as indicated. (**f**) Myoblast fusion and myotube growth are impaired by elastase treatment. C2C12 myoblasts were cultured in growth medium until confluent then switched to differentiation medium in the presence/absence of 0.15 U/mL elastase as indicated and maintained for an additional 4 days prior to fixation and immunostaining to detect myosin heavy chain (MyHC) and nuclei (DAPI). (**g,h**) Quantification of (**f**) where the fusion index is the percentage of MyHC + cells in myotubes over the total number of MyHC + cells (**g**), while the myotube area (**h**) is calculated as total MyHC + area per image. Plots are averages of 10–15 technical replicates across 4 biological replicates (n > 40 per data-point). NT = non-treated, Elast = 0.15 U/mL elastase. Error bars are S.E.M. **p < 0.01. Scale bars are: 30 μm in (**a,b**) and 100 μm in (**f**).

Reduced cell numbers could be caused by induction of cell death or lack of proliferation. To test whether reduced numbers were caused by a lack of proliferation we immunostained myoblasts treated with elastase and untreated to detect the cell cycle marker Ki67. Moderate levels of Ki67 are present in the nucleus of dividing cells during any phase of the cell cycle but dramatically increase during M-phase (Fig. 7a). The total numbers of Ki67 + cells was decreased in elastase-treated cells compared to non-treated cells (Fig. 7b). Moreover, the number of mitotic cells, identified by increased Ki67 staining and nuclear morphology consistent with M-phase, was dramatically reduced in elastase-treated cultures compared to non-treated cultures in a dose-independent manner (Fig. 7c).

The chronic presence of elastase in dystrophic muscle and especially in association with major components of the satellite cell niche such as laminin and collagen IV, can lead to impaired myogenesis via decreasing myoblast survival and proliferation. To test whether elastase activity affects also myoblast cell fate, we cultured myoblasts in growth medium in the presence/absence of elastase and after a 24 hour-long treatment we detected MyoD1 via immunostaining. Myoblasts exposed to elastase showed a dramatic reduction in MyoD1 protein levels (Fig. 7e) compared to non-treated myoblasts (Fig. 7d). Consistent with a reduction in MyoD1 levels, myoblast differentiation into myotubes was also impaired (Fig. 7f). We investigated the response of differentiating myoblasts to concentrations of elastase that did not cause cell death in the presence of 3% horse serum (0.15 U/mL elastase). Although the percentage of cells that differentiated was unchanged by elastase treatment, the differentiated cells had lower fusogenic capacity (Fig. 7f,g) and the myotubes generated by elastase-treated myoblasts were smaller (Fig. 7f,h). The effect on myoblast fusion and myotube area was independent on the substrate to which cells adhered suggesting that it was mediated by an effect of elastase on secreted or serum factors or cell receptors rather than on the ECM.

### Elastase reduces primary myoblast growth and differentiation

**Figure 8:**
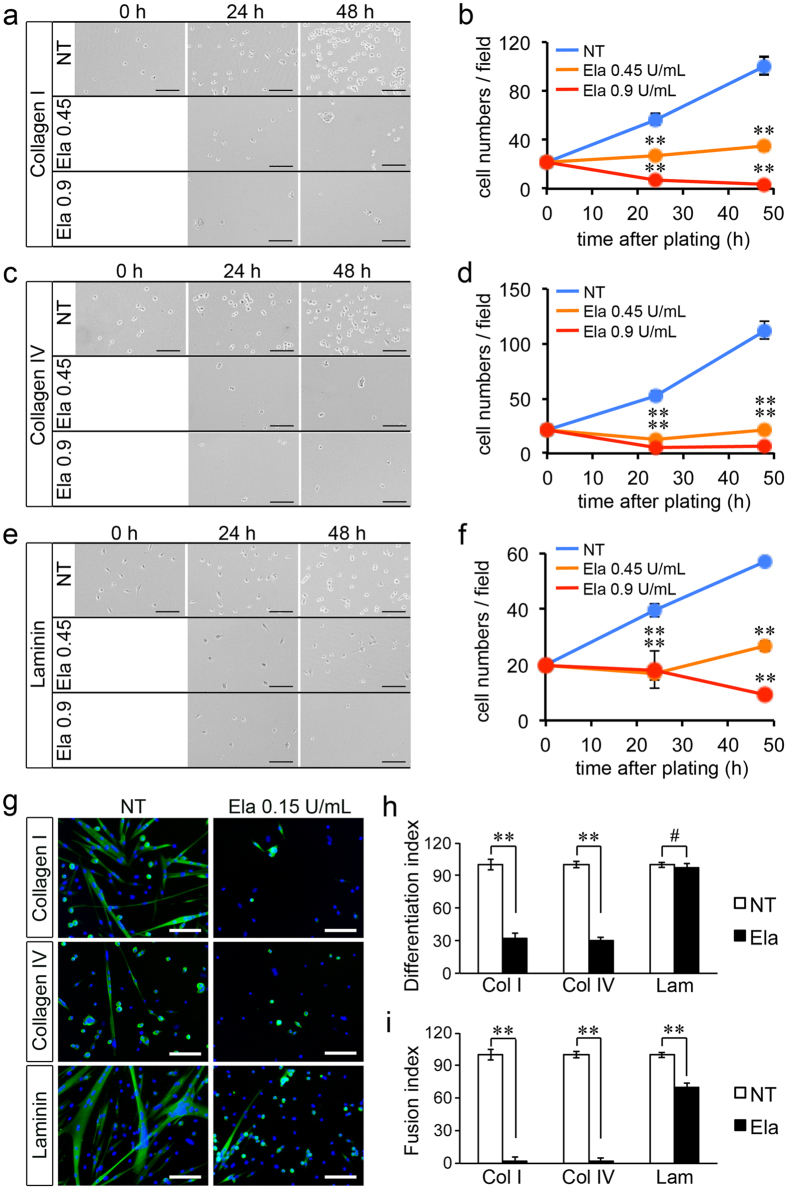
Elastase treatment impairs primary myoblast growth and differentiation rates. (**a,c,d**) Representative images of primary myoblasts seeded on collagen type I (**a**), collagen type IV (**c**) or laminin (**e**) and either non-treated or treated with elastase (0.45 U/mL and 0.9 U/mL) for 1 and 2 days. (**b**,**d**,**f)** Quantification of (**a,c,e**), respectively. For quantification, 10 images per experiment across 3 independent experiments (N = 30 images) were scored for cell numbers, averaged and plotted as a function of time. (**g**) Representative images of primary myoblasts maintained in differentiation medium for three days then immunostained to detect myosin heavy chain (MyHC, green) and DAPI (blue). (**h**) The differentiation index was calculated as the percentage of MyHC + cells over the total number of DAPI + cells, then for each culture condition (Col I, Col IV and Lam) the differentiation index of non-treated cells was set to 100% and the differentiation index of treated cells expressed as a fraction of 100 in order to average different experiments. Ten images per experiment across 3 independent experiments were analysed (N = 30). (**i**) The fusion index was calculated as the percentage of MyHC + cells in myotubes over the total number of MyHC + cells, then for each culture condition (Col I, Col IV and Lam) the fusion index of non-treated cells was set to 100% and the fusion index of treated cells expressed as a fraction of 100 in order to average different experiments. Ten images per experiment across 3 independent experiments were analysed (N = 30). Error bars are S.E.M.; **p < 0.01; ^#^p > 0.05. Scale bars are 100 μm.

To test the role of elastase in myogenesis in a more physiological context we obtained primary myoblasts from hind limb mouse muscles, plated them on either collagen type I, collagen type IV or laminin and then treated them with either elastase or vehicle. We chose as a reference the two highest concentrations of elastase that yielded significant differences in proliferation and survival of C2C12 cells, and increased them to take into account the 50% increase in serum (and therefore 50% increase in serine protease inhibitors found in serum) that is used in primary myoblast cultures compared to C2C12 cell cultures (15% vs 10% serum, see *Methods* section for details). When primary myoblasts were treated with elastase they showed a dramatic and dose-dependent reduction in growth rates compared to non-treated myoblasts (Fig. 8a–f). Interestingly, we noted that myoblasts plated on laminin proliferated less than myoblasts plated on collagen type I or collagen type IV after a 2 day-long expansion on gelatin. This was accompanied by a modestly reduced efficacy of elastase in decreasing the growth rate when cells were plated on laminin compared to when cells were plated on either collagen type I or collagen type IV. Nonetheless, the effect of elastase on primary myoblats plated on any of the three substrates was significant and the effect sizes large. Thus, primary myoblasts were highly sensitive to elastase treatment under all three culture conditions suggesting that *in vivo*, elastase might be detrimental to myoblasts regardless of their anatomical location, and significantly contribute to the impaired myogenesis observed in dystrophic muscle.

We then further studied how elastase affects myogenesis by testing its effect on primary myoblast differentiation. For these experiments we used the same concentration of elastase that we had previously used for the C2C12 myoblast differentiation experiments, as the concentration of serum in the differentiation medium was the same for C2C12 and primary myoblasts (3%). Addition of elastase to differentiating primary myoblasts caused a dramatic reduction in differentiation and fusion when cells were seeded on collagen type I or collagen type IV while differentiation of primary cells seeded on laminin was not affected by elastase (Fig. 8g,h). In contrast, the fusion index was decreased in myoblasts treated with elastase under all three culture conditions (Fig. 8g,i).

Our data together indicate that the chronic presence of elastase activity in the extracellular environment of dystrophic muscle might impair myogenesis at multiple levels and via multiple mechanisms.

## Discussion

In this paper we show that: (i) we have developed a method to detect changes in the soluble component of the muscle ECM using label-free shotgun proteomics; (ii) the levels of neutrophil elastase protein and activity are increased in dystrophic muscle and become progressively higher over time as the disease progresses in combination with loss of regenerative potential; (iii) elastase activity is toxic to muscle progenitors and impairs myogenesis *ex vivo*.

ECM accumulation and other changes in the muscle extracellular environment are a hallmark of muscular dystrophy and can affect the function of the myofibres themselves, but also of other cell types present in the muscle tissue^1^,^21^,^22^,^34^,^35^. In remarking the importance of fibrosis in the pathophysiology of DMD, recent studies have shown that treatments aimed at reducing fibrosis produce an overall amelioration of the dystrophic phenotype^36^,^37^. Discovery proteomics through mass spectrometry offers an unbiased approach to the identification of disease-associated changes in the extracellular environment, however discovery proteomics of skeletal muscle is challenging due to the extremely high abundance of a restricted repertoire of intracellular proteins in muscle fibres^29^. These very abundant proteins are mainly components of the contractile apparatus and enzymes involved in energy metabolisms (mostly glycolysis and TCA cycle) but also common contaminants such as cytoskeletal and ribonuclear proteins^29^. Thus, it is difficult to profile by mass spectrometry the extracellular compartment of skeletal muscle, which is largely under-represented compared to the intracellular fraction. Several groups have analyzed dystrophic muscle before using mass spectrometry approaches and contributed to the identification of potential pathogenic mechanisms. However, the fraction of extracellular proteins detected was relatively small and often limited to structural proteins such as collagens, decorin, dermatopontin and periostin^38^,^39^,^40^,^41^,^42^,^43^,^44^. Our approach allowed us to confirm most of the changes in extracellular matrix proteins that were previously identified by other groups, such as a dramatic increase in periostin^37^,^39^, while also identifying changes in extracellular proteins that were not previously detected and, most importantly, identify the regulation of serine protease activity as a key function that is altered in dystrophic muscle compared to wild type muscle.

Although a pathogenic role for some of the serine proteases in the coagulation cascade had been previously shown in muscular dystrophy^45^,^46^, our findings suggest a higher level of complexity for altered serine protease activity in the pathogenesis of muscular dystrophy than previously appreciated. We decided to focus on elastase because the abundance of the elastase inhibitor Serpinb1a was the most significantly different between wild type and dystrophic mice at both ages examined. Serpins are endogenous serine protease inhibitors usually produced and secreted to protect the tissue from the activity of proteases, in this case elastase^47^,^48^,^49^. Indeed we found that elastase, which is normally present only around blood vessels and nerves in healthy muscle tissue, was increased in the myofibre interstitium of dystrophic muscle. This is likely due to the presence of chronic inflammation and consequent presence of elastase-secreting cells, especially neutrophils. It is known that in the context of inflammation serine proteases can remain active despite the presence of Serpins since the large amount of proteases released by immune cells overwhelms and inhibits Serpins, ultimately inactivating them^47^. Thus, we sought to investigate further whether elastase activity was altered in dystrophic muscle and we found that is increased in the muscle of older dystrophic mice, whose muscle regenerative capacity is impaired, but not in young mice, in which muscle regeneration is still efficient. Since elastase is involved in ECM remodeling, regulation of cytokines and other signaling molecules^50^,^51^,^52^,^53^,^54^,^55^, the increase in elastase activity that we document here could be one of many factors that lead to loss of regenerative capacity in dystrophic muscle. Indeed we show that elastase treatment of myoblast cultures impairs cell survival, proliferation and differentiation. Impairment of C2C12 cell survival is partly dependent on the nature of the substrates to which the cells adhere and correlates with the kinetics of elastase-mediated cleavage of such substrates, suggesting that substrate degradation might be one of the mechanisms mediating elastase effect on C2C12 cell survival. Interestingly, impairment of primary myoblast cell growth depends on the substrate to a lesser extent and in a different way from C2C12 cells: while C2C12 cells are more resistant to elastase when seeded on collagen type I, primary myoblasts are slightly more resistant to elastase when seeded on laminin, though only marginally. This suggests that substrate degradation is only partly involved in mediating elastase effect on myoblast growth. For example, the two cell types could adhere to the same substrate in different ways and be differentially affected by degradation of the same substrate. Since the overall growth of primary myoblasts was dramatically affected by elastase in similar ways across all three culture conditions examined, it is plausible that in primary cultures the effect of elastase on factors other than the substrate is more pronounced than the effect on the substrate. Moreover, the effect of elastase on C2C12 cell proliferation, fusion, MyoD1 expression, and myotube growth do not appear to be substrate-dependent, further supporting the idea that substrate-independent mechanisms also mediate elastase effects on myogenesis. Indeed, a key role for elastase in cleaving and either activating or inactivating cytokines and growth factors has been shown^50^,^51^,^52^,^53^,^54^,^55^. Possible candidates include pro-inflammatory cytokines such as IL-6 and IL-1β whose expression and secretion are promoted by neutrophil elastase^56^,^57^,^58^. Both IL-6 and IL-1β are produced by immune cells in injured muscle but also by myoblasts in culture, and increased levels of IL-6 and IL1 β have been associated with muscle wasting and impaired myogenesis^59^,^60^,^61^. In addition to cytokines, the effect of elastase on growth factor release from the ECM should also be considered. For example, in the lung, elastase promotes release of TGF-β from the matrix and its activation^62^. TGF-β inhibits myoblast differentiation^63^,^64^ and thus its release during differentiation might be one of the mechanisms contributing to elastase-induced impairment of terminal differentiation and fusion. Lastly, elastase might also affect myogenesis via cleavage and activation of the thrombin receptors, which are normally cleaved by thrombin but can also be cleaved and activated by elastase^65^,^66^, leading to delayed myogenin expression and myoblast differentiation^67^.

Neutrophils are important regulators of inflammation and ECM remodeling. In response to tissue injury neutrophils invade the damaged tissue and promote tissue repair mainly by clearing the tissue debris via phagocytosis and by promoting macrophage infiltration. During muscle regeneration neutrophils play a double role: while neutrophil activity exacerbates muscle injury via secretion of cytolytic and cytotoxic molecules, such as proteases, it also promotes muscle repair via clearing of damaged muscle debris and attraction of macrophages, which in turn sustain satellite cell activation and proliferation^23^. During acute injuries, the wave of neutrophil infiltration only lasts 1–2 days, then it resolves during myoblast proliferation and differentiation^6^. In contrast, we show here that the numbers of neutrophil infiltrates in dystrophic muscle increase over time and that at least one of the proteases abundantly secreted by neutrophils in dystrophic muscle, elastase, increases as well and is toxic to myoblasts in culture. Thus, we conclude that the chronic presence of neutrophils in dystrophic muscle further exacerbates muscle injury both directly, via secretion of cytotoxic molecules, and indirectly by impairing muscle regeneration. Consistently, it has been shown that neutrophil depletion in the *mdx* model of DMD leads to an amelioration of the dystrophic phenotype^68^.

Several selective inhibitors of neutrophil elastase and other serine proteases have been developed and clinically tested as safe and thus, our discovery of a pathogenic role for elastase activity in muscular dystrophy has important translational relevance.

## Methods

### Mice

*Mdx*^*4cv*^ mice, generated on the C57Bl/6 background, were obtained from Jackson Laboratories, housed in a pathogen-free facility at the University of Liverpool, UK and used in accordance with the Animals (Scientific Procedures) Act 1986 and the EU Directive 2010/63/EU and after local ethical review and approval by Liverpool University’s Animal Welfare and Ethical Review Body (AWERB). Age-, sex- and background-matched wild type (C57Bl/6) controls were purchased from Charles River UK and housed for at least 2 weeks in the same facility as *mdx*^*4cv*^ mice before sacrifice.

### Muscle sample collection

Muscles from mice belonging three different age groups (3, 5 and 7.5 months of age) were initially processed and analysed. Of these, the 3- and 7.5-month age groups were selected for further analysis. Gastrocnemius muscles of male wild type and dystrophic mice were dissected and immediately subjected to a brief digestion with 400 U/mL collagenase type I (*Worthington*). Since wild type and dystrophic muscles of different ages are also different in size and weight, preliminary experiments were carried out to identify the minimum incubation time required to allow mechanical separation of myofibre groups. This time was directly related to muscle weight and resulted in: 5 minutes for 3 month old wild type muscle, 7 minutes for 3 month-old dystrophic muscle, 10 minutes for 7.5 months-old wild type muscle and 15 minutes for 7.5 months-old dystrophic muscle. The collagenase solution was then removed, the muscle washed in ice-cold Dulbecco-modified Eagle’s Medium (DMEM, *Life Technologies*) and the bundles of myofibres separated mechanically with a pair of fine tweezers under a microdissection microscope. This process exposed the endomysium and the basal lamina, which are the natural environment in which muscle progenitors are found, to the subsequent trypsin digestion. Separated bundles of myofibres were then incubated at 37 °C with 1 μg/mL mass spectrometry-grade trypsin (Trypsin Gold, *Promega*) in Phosphate Buffered Saline (PBS). After 40 minutes myofibre bundles were centrifuged and the supernatant containing trypsin-generated fragments of extracellular proteins and intracellular proteins leaked from damaged myofibres were collected and immediately frozen. Four or five biological replicates per genotype and per age group were collected.

### Muscle sample processing

Samples were thawed on ice and centrifuged at 17,200 ×g for 5 minutes. A 200-fold dilution was made for protein assay using a Bradford assay reagent (Coomassie Plus, *Thermo Scientific Pierce*). A volume of sample equivalent to 50 μg of protein was diluted to a volume of 80 μL with 25 mM ammonium bicarbonate. Samples were then age and genotype randomized for reagent addition: 5 μL of 1% (w/v) Rapigest (*Waters*) in 25 mM ammonium bicarbonate was added and the samples heated at 80 °C for 10 minutes. Samples were reduced by the addition of 5 μL of 60 mM DTT in 25 mM ammonium bicabonate and heated at 60 °C for 10 minutes. Samples were cooled and 5 μL of 178 mM iodoacetamide in 25 mM ammonium bicarbonate was added and samples incubated at room temperature in the dark for 30 minutes. Trypsin 5 μL (1 μg, Trypsin Gold, *Promega*) was added and the samples incubated at 37 °C overnight. Samples were acidified by the addition of 1 μL trifluoracetic acid (TFA) and after incubation at 37 °C for 45 minutes samples were centrifuged at 17,200 × g for 30 minutes and the clarified supernatants transferred to fresh low-binding tubes. To check for complete digestion each sample was analyzed pre- and post-acidification by SDS-PAGE.

### High resolution LC-MSMS analysis

A 5-fold dilution of digest (100 ng protein equivalent) was injected on-column and chromatographed over a 50 minute gradient using a method whereby, following a survey scan at 70,000 resolution, the top 10 most abundant peptide ions are fragmented and measured at high resolution (35,000) in the Orbitrap analyzer to a mass accuracy of 0.01 Da.

### LC separation

All peptide separations were carried out using an Ultimate 3000 nano system (Dionex, *Thermo Fisher Scientific*). For each analysis the sample was loaded onto a trap column (Acclaim PepMap 100, 2 cm × 75 μm inner diameter, C18, 3 μm, 100 Å) at 5 μL/min with an aqueous solution containing 0.1% (v/v) TFA and 2% (v/v) acetonitrile. After 3 min, the trap column was set in-line with an analytical column (Easy-Spray PepMap® RSLC 15 cm × 75 μm inner diameter, C18, 2 μm, 100 Å (Dionex). Peptide elution was performed by applying a mixture of solvents A and B. Solvent A was HPLC grade water with 0.1% (v/v) formic acid, and solvent B was HPLC grade acetonitrile 80% (v/v) with 0.1% (v/v) formic acid. Separations were performed by applying a linear gradient of 3.8% to 50% solvent B over 30 min at 300 nL/min followed by a washing step (5 min at 99% solvent B) and an equilibration step (15 min at 3.8% solvent B).

### Mass spectrometry

The Q-Exactive instrument was operated in data dependent positive (ESI+) mode to automatically switch between full scan MS and MS/MS acquisition. Survey full scan MS spectra (m/z 300–2000) were acquired in the Orbitrap with 70,000 resolution (m/z 200) after accumulation of ions to 1 × 10^6^ target value based on predictive automatic gain control (AGC) values from the previous full scan. Dynamic exclusion was set to 20 s. The 10 most intense multiply charged ions (z ≥ 2) were sequentially isolated and fragmented in the octopole collision cell by higher energy collisional dissociation (HCD) with a fixed injection time of 120 ms and 35,000 resolution. Typical mass spectrometric conditions were as follows: spray voltage: 1.9 kV, no sheath or auxillary gas flow; heated capillary temperature: 250 °C; normalized HCD collision energy 30%. The MS/MS ion selection threshold was set to 1 × 10^4^ counts and a 2 m/z isolation width was set.

### Label-free quantification

The data were processed with ProgenesisQI (version 4, *Nonlinear Dynamics*, Newcastle upon Tyne, UK). Samples were automatically aligned according to retention time. Default peak picking parameters were applied and features (peptides) with charges from 2+ to 7+ were retained. Ion abundance measurements were made in all LC-MS runs and the data was normalised to make comparisons between runs. Database searching was performed using Mascot (*Matrix Science*, London, UK). A Mascot Generic File, created by ProgenesisQI, was searched against the mouse UniProt database. A fixed carbamidomethyl modification for cysteine and variable oxidation modification for methionine were specified. A precursor mass tolerance of 10 ppm and a fragment ion mass tolerance of 0.01 Da were applied. The results were then filtered to obtain a peptide false discovery rate assigned to the Mascot search. Proteins were quantified using unique peptides and protein abundance was calculated from the sum of all the unique peptide ion abundances corresponding to that protein. Protein quantification was further validated by using a separate software package, the mzqLibrary^28^, which yielded results comparable to the results obtained with ProgenesisQI.

### Statistical analysis of proteomics data

Analysis of variance (ANOVA) was applied across genotypes and across age groups to identify proteins that significantly change. Proteins that changed with a p-value smaller than 0.05 were considered of moderate statistical significance. Proteins that changed with a q-value (Bonferroni-corrected p-value) smaller than 0.05 were considered of strong statistical significance.

### Immunofluorescence

Tissue samples were dissected and immediately frozen in liquid nitrogen-cooled isopentane. For all immunofluorescence staining except with anti-Serpinb1, sections were fixed with 4% paraformaldehyde (PFA) in PBS for 10 minutes at room temperature. After three washes in PBS, sections were permeabilzed with PBS + 0.2% TritonX100 (PBST) followed by a blocking step with 3% bovine serum albumin (BSA) prior to overnight incubation with primary antibodies at 4 °C in PBS +1% BSA. For immunostaining with the mouse anti-Serpinb1 antibody, sections were not fixed, 1% mouse serum was added to 3% BSA in the blocking step and 0.03% mouse serum was added to 1% BSA to the primary antibody incubation step. After 1 wash in PBST followed by two washes in PBS, sections were blocked again for 30 minutes with 10% horse serum (supplemented with 1% mouse serum for Serpinb1 immunostaining). Secondary antibody incubation was carried out for 1 h at room temperature in 5% horse serum (supplemented with 1% mouse serum for Serpinb1 immunostaining), followed by 1 wash in PBST, 1 wash in PBS + DAPI 2 μg/mL, 2 washes in PBS and mounting. For immunofluorescence of cells, C2C12 cells cultured and treated as described below were fixed with 4% PFA for 10 minutes at room temperature then washed 3 times with PBS, permeabilzed 10 minutes at room temperature with PBST and blocked for 1 h at room temperature with 10% horse serum. Primary antibody incubation was carried out overnight at 4 °C in PBS + 1% horse serum and then the same protocol as above was followed for the second blocking and the secondary antibody incubation. The antibodies used were: rabbit anti-Ly6G (*Bioss*) at 1:100, rabbit anti-neutrophil elastase (*Bioss*) at 1:100, mouse anti-Serpinb1 (*Sino Biologicals*) at 1:100, rat anti-laminin alpha-2 (*Sigma*) at 1:150 and Rabbit anti-laminin (*Sigma*) at 1:200, mouse anti-MyoD1 (*BD Biosciences*) at 1:100, rabbit anti-Ki67 (*Abcam*) 1:400, mouse anti-myosin heavy chain (MF20 clone, *DSHB*) at 1:100. Secondary antibodies made in donkey and conjugated with Alexa 555 and Alexa 488 (*Molecular Probes*) were used at 1:500 dilution. Nuclei were detected via DAPI (*Life Technologies*) staining. Vectashield (*Vector Laboratories*) was used as mounting medium.

### Image analysis

Images were acquired on an EVOS FL imaging system (*Life Technologies*) with a 10× or a 40× objective and consistent imaging parameters. Post-processing was performed using a bespoke script for the Open Source image processing program Fiji (http://fiji.sc). Briefly, RGB images were split into channels depicting nuclei and either MyHC (Fig. 8) or Laminin (Fig. 1), which were then thresholded based on either the Li or Triangle method. Large inseparable areas of nuclei (after watershed filtering) were removed (maximum size cutoff of 900 pixels) and the remaining objects were counted automatically. Myotube Area, Differentiation Index (number of nuclei in MyHC + cells divided by total number of nuclei) and Fusion index (number of cells in myotubes divided by total number of MyHC + cells) were calculated using logical binary arithmetic on thresholded masks of the MyHC positive myotubes and nuclei, followed by object counting and measurement as required.

### Western blot

Quadriceps from wild type and dystrophic mice were homogenized in RIPA buffer (150 mM NaCl, 50 mM Tris-HCl, pH 7.5, 1.0% IGEPAL, 0.1% SDS, 0.5% sodium deoxycholate) supplemented with protease inhibitor cocktail (Complete, *Roche*) and phosphatase inhibitors (1 mM Na_3_VO_4_ + 1 mM NaF) using an UltraTurrex homogenizer followed by incubation on ice for 30 minutes and then cleared by centrifugation at 13,000 rpm for 10 min at 4 °C. Western blot was performed as previously described^69^. The antibodies used were: rabbit anti-Serpinb1 (Sigma Prestige) at 1:500; rabbit anti-neutrophil elastase (*Bioss*) at 1:1000 and anti-rabbit HRP-conjugated secondary antibody (*Santa Cruz Biotechnology*) used at 1:10,000. HRP activity was visualized using the ECL Clarity^TM^ (*BioRad*) system and recorded on a LAS 4000 (*GE*) gel doc system.

### Enzymatic activity kinetics measurement

Quadriceps from wild type and dystrophic (*mdx*^*4cv*^) mice at 3 or 7.5 months of age were dissected and homogenized in 150 mM NaCl, 50 mM Tris-HCl pH 7.5 in ice then incubated with/without protease inhibitors (Complete, *Roche*; Elastatinal, *Sigma*; Sivelestat, *Enzo Scientific*) for 1 hour at room temperature prior to being incubated for 3 hours at 37 °C in a temperature controlled plate reader (BMG *Labtech*) with 5 nM Suc-Ala-Ala-Ala-AMC (*Peptanova*). The absorbance at 450 nm was read every 15 minutes and recorded.

### Cell culture

C2C12 myoblasts (kindly donated by Dr Olwin, University of Colorado, Boulder) were maintained in growth medium (DMEM +10% fetal bovine serum +1% L-glutamine +1% penicillin/streptomycin) at a confluence of 40–70% (thus passaged every 48 hours). To study the effect of elastase on C2C12 myoblasts, cells were seeded on 12 multi-well plates (*Corning-Costar*) coated with either collagen I from rat tail (*Life Technologies*), collagen type IV from human placenta (*Sigma*) or laminin from Engelbreth-Holm-Swarm murine sarcoma basement membrane (*Sigma*) in growth medium. For elastase treatment of proliferating cells, cultures were maintained for 1–2 days until a confluence of 50–60% was reached then elastase (*Sigma*) was added and maintained for an additional 1 or 2 days. To induce differentiation, myoblasts were let grow to 90% confluence then the medium was switched to differentiation medium (DMEM + 3% horse serum + 1% L-glutamine + 1% penicillin/streptomycin) and maintained for 4 days. For elastase treatment of differentiating cells, elastase was added with the differentiation medium. The variable activity of different batches of elastase was taken into account. Moreover we noticed variability across individual vials received from the manufacturer with 2 vials out of 15 used in total showing virtually no activity. In order to take this variability into account, biological replicates were independent experimental replicates carried out using different vials of elastase. The same batches of fetal bovine serum and horse serum were used in all experiments.

Primary myoblasts were obtained as previously described^69^. Briefly: hind limb muscles of C57Bl/6J mice were dissected, minced with scissors and digested with collagenase type I (*Worthington*) then centrifuged and filtered through a 40 μm cell strainer to isolate mono-nucleated cells. After two subsequent pre-plating steps on gelatin-coated tissue culture dishes (2 h + 1 h) the floating cells were plated on gelatin-coated dishes in growth medium (F12 + 0.4 mM CaCl_2_ + 15% horse serum + 1% penicillin/streptomycin + 2 mM glutaMAX + 5 nM FGF2) for two days prior to being collected and re-plated on either collagen type I, collagen type IV or laminin. Myoblast cell purity was >95%. Differentiation was induced by replacing the growth medium with differentiation medium (F12 +0.4 mM CaCl_2_ +3% horse serum +2 mM glutaMAX +1% penicillin/streptomycin).

### Flow Cytometry

Cells were collected 16 hours after treatment with elastase, centrifuged, resuspended and incubated with Annexin-V-FITC and DAPI for 15 minutes on ice prior to analysis on a FACSCanto (*Becton Dickinson*). The gating scheme for quantification of Annexin-V+ and DAPI+ cells is shown in Fig. S7a. Samples were run entirely in order to obtain an estimate of the total numbers of detached cells.

### Statistical analysis

For all experiments using cells the data collected distributed normally and therefore a two-tailed Student’s t-test was applied to calculate statistical significance. A p-value smaller than 0.05 was considered statistically significant.

## Additional Information

**How to cite this article**: Arecco, N. *et al*. Elastase levels and activity are increased in dystrophic muscle and impair myoblast cell survival, proliferation and differentiation. *Sci. Rep*. **6**, 24708; doi: 10.1038/srep24708 (2016).

## Supplementary Material

Supplementary Information
