# Supplementary material for: Elastase levels and activity are increased in dystrophic muscle and impair myoblast cell survival, proliferation and differentiation
Source: Sci Rep. 2016 May 31;6:24708. doi: 10.1038/srep24708 (PMC4886533; doi:10.1038/srep24708)
Supplement: Supplementary Information [file srep24708-s1.pdf]

Elastase levels and activity are increased in dystrophic muscle and impair myoblast cell survival, proliferation and differentiation

N. Arecco[1,2,3], *C.J. Clarke*[1,2], *F.K. Jones*[1,2], *D. Simpson*[1,4], *D. Mason*[1,5], *R.J. Beynon*[1,4], *A. Pisconti*[1,*]

[1]Department of Biochemistry, Institute of Integrative Biology, University of Liverpool, Crown Street, Liverpool L69 7ZB, UK. [4]Centre for Proteome Research, University of Liverpool, Liverpool L69 7ZB, UK. [5]Centre for Cell Imaging, University of Liverpool, Crown Street, Liverpool L69 7ZB, UK.

SUPPLEMENTARY INFORMATION

[2] These two authors, listed in alphabetical order, contributed equally to this work

[3] Current address: European Molecular Biology Laboratory (EMBL), Meyerhofstraße 1, 69117 Heidelberg, Germany

[*] Corresponding author: pisconti@liverpool.ac.uk, Tel: +44 (0)151 795 4428

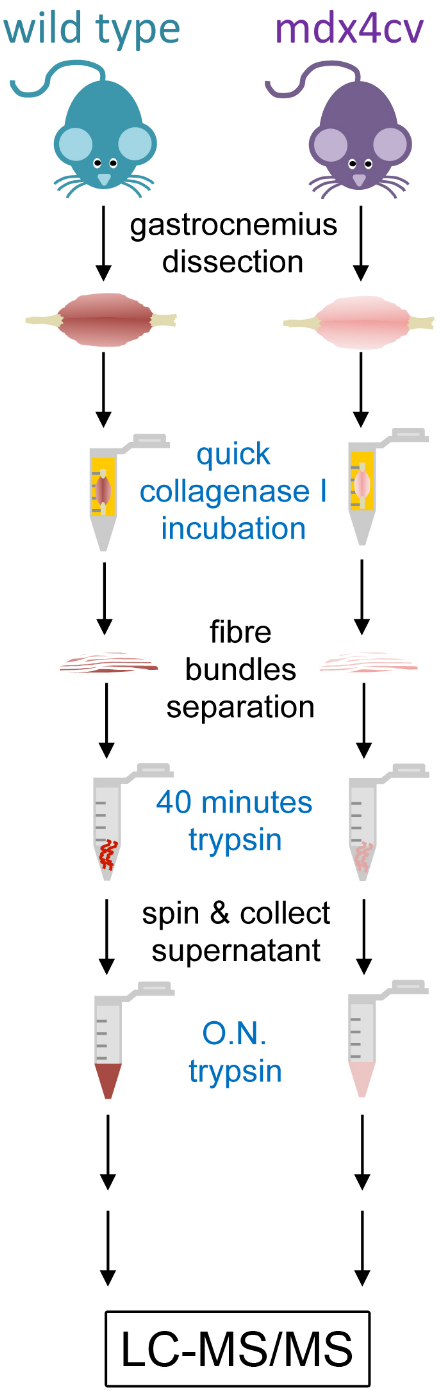

Supplementary Figure S1: Schematic diagram of the sample preparation workflow prior to processing for mass spectrometry.

a

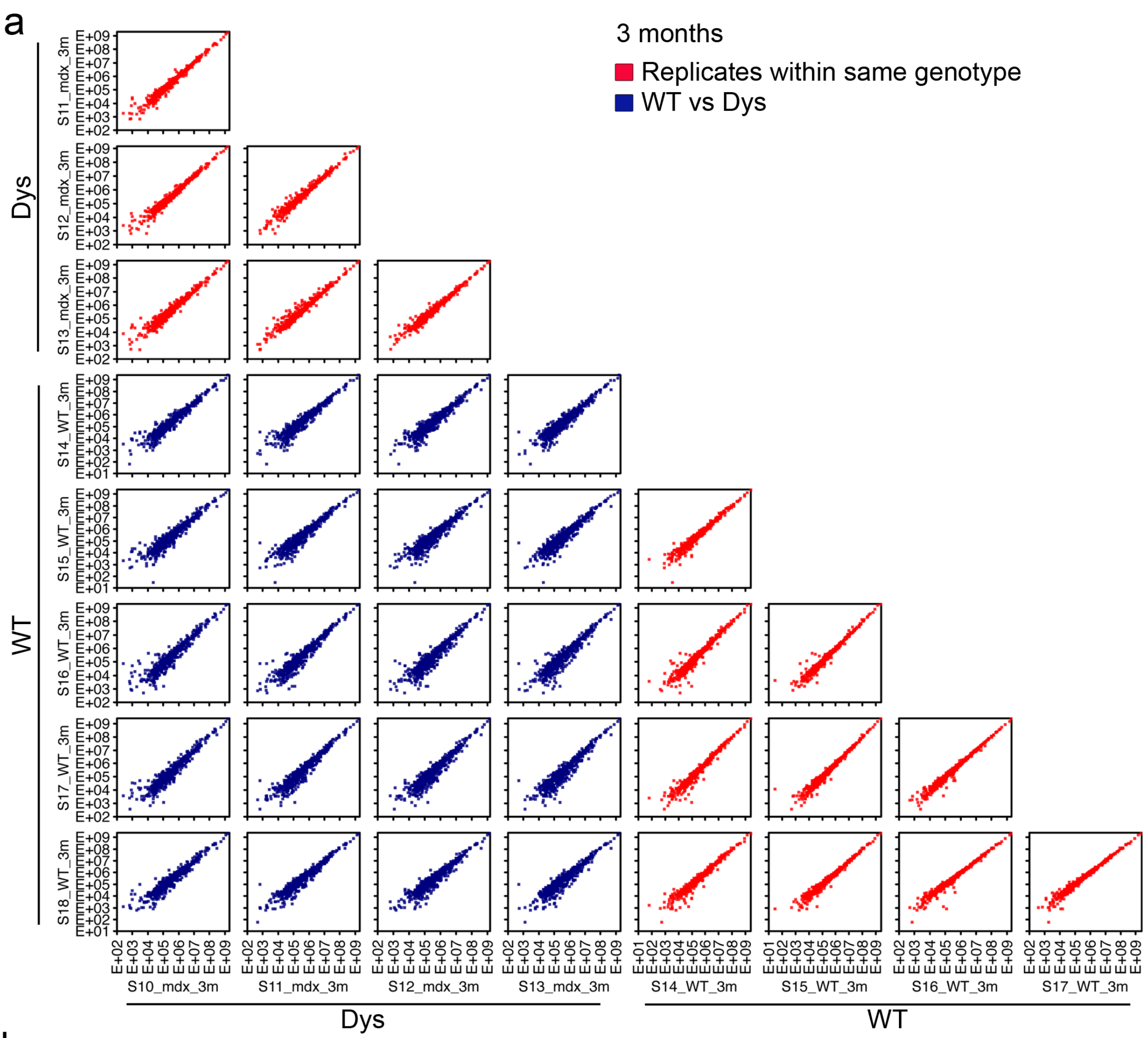

b

		S10	S11	S12	S13	S14	S15	S16	S17
Dys	S11	0.9915							
Dys	S12	0.9892	0.9795						
Dys	S13	0.9866	0.9868	0.9931					
WT	S14	0.9542	0.9619	0.9507	0.9394				
WT	S15	0.9621	0.9766	0.9534	0.9532	0.9941			
WT	S16	0.9733	0.9928	0.9594	0.9747	0.9563	0.9770		
WT	S17	0.9679	0.9847	0.9582	0.9659	0.9830	0.9961	0.9880	
WT	S18	0.9681	0.9907	0.9524	0.9700	0.9555	0.9785	0.9970	0.9904
		Dys	Dys	Dys	Dys	WT	WT	WT	WT

<u>Supplementary Figure S2</u>: Quality control of proteomics data for the 3 months age group. **a)** Correlation matrix for all samples (wild type and dystrophic) collected from 3 month-old mice. **b)** Correlation coefficients of the plots shown in (a). Scatter plots and correlation coefficients for replicates within the same genotype (wild type versus wild type and dystrophic versus dystrophic) are shown in red while scatter plots and correlation coefficients for wild type replicates versus dystrophic replicates are shown in blue.

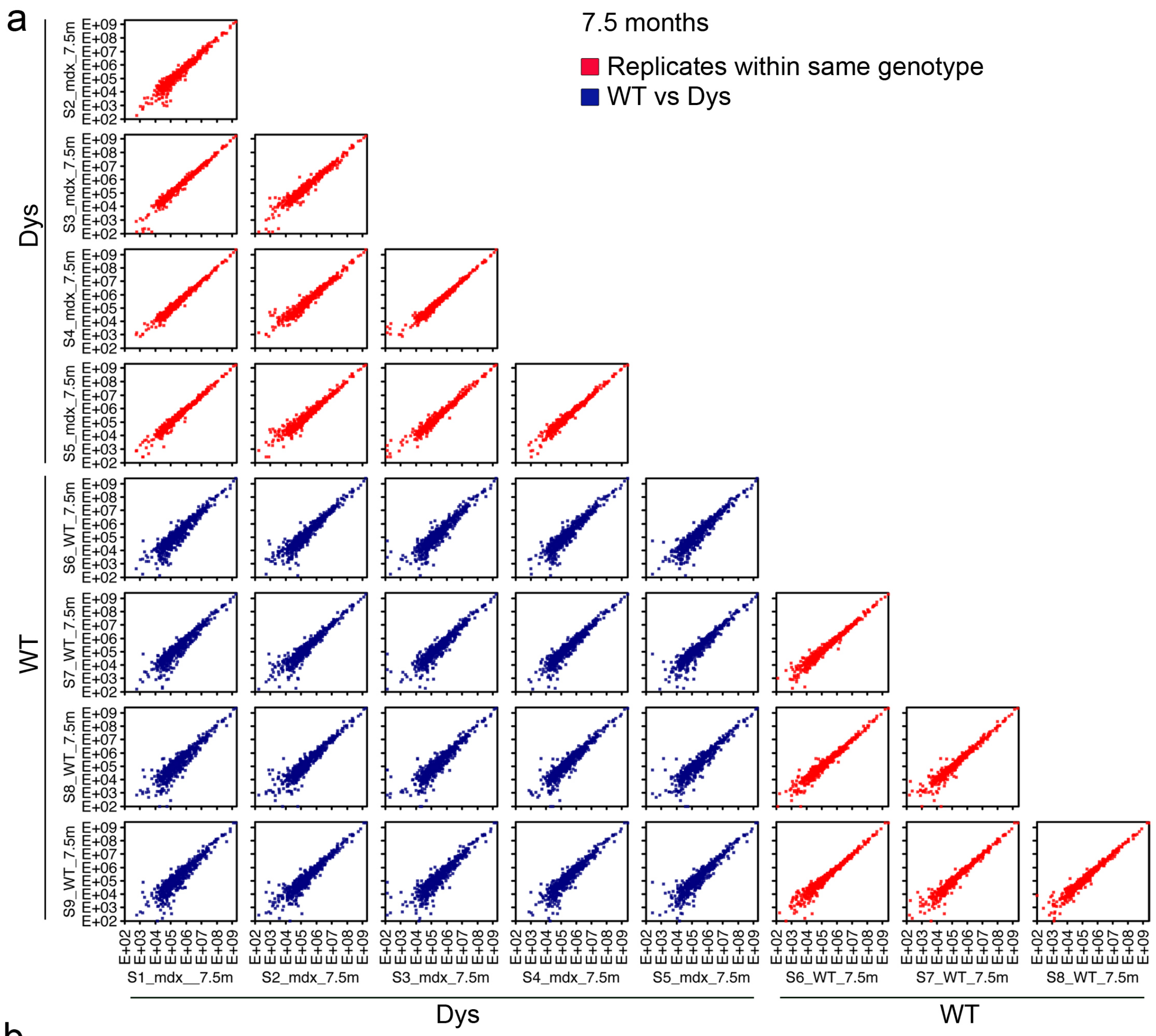

b

		S1	S2	S3	S4	S5	S6	S7	S8
Dys	S2	0.9839							
Dys	S3	0.9974	0.9896						
Dys	S4	0.9880	0.9853	0.9861					
Dys	S5	0.9921	0.9885	0.9927	0.9763				
WT	S6	0.9450	0.9655	0.9428	0.9776	0.9405			
WT	S7	0.9640	0.9828	0.9709	0.9914	0.9604	0.9894		
WT	S8	0.9526	0.9771	0.9558	0.9700	0.9655	0.9818	0.9731	
WT	S9	0.9260	0.9630	0.9322	0.9398	0.9510	0.9601	0.9461	0.9917
		Dys	Dys	Dys	Dys	Dys	WT	WT	WT

<u>Supplementary Figure S3</u>: Quality control of proteomics data for the 7.5 months age group.

a) Correlation matrix for all samples (wild type and dystrophic) collected from 7.5 month-old mice.

b) Correlation coefficients of the plots shown in (a). Scatter plots and correlation coefficients for replicates within the same genotype (wild type versus wild type and dystrophic versus dystrophic) are shown in red while scatter plots and correlation coefficients for wild type replicates versus dystrophic replicates are shown in blue.

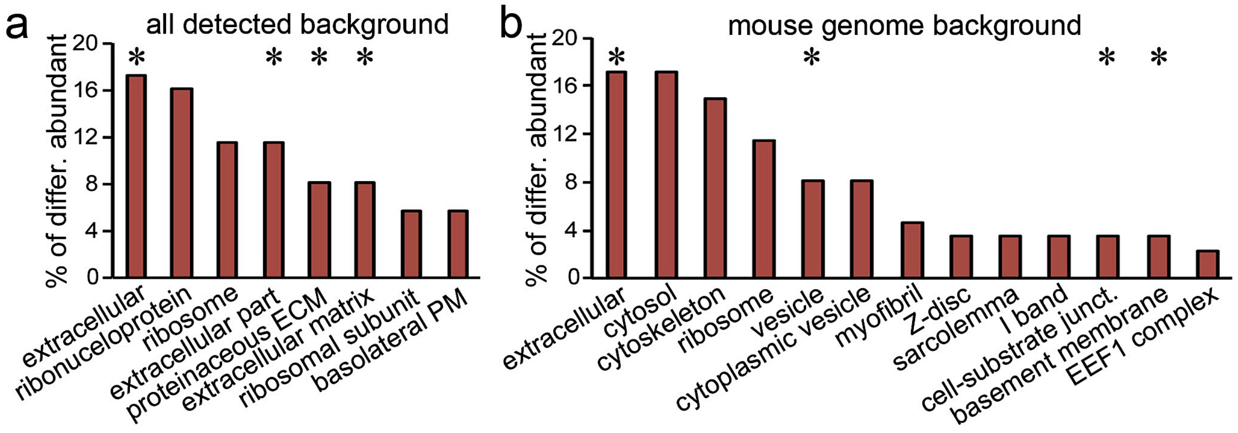

Supplementary Figure S4: The proteomics method developed enriches muscle sample preparations with extracellular proteins. a-b) Mapping of all proteins found differentially abundant between wild type and dystrophic muscle to the GO category *Cellular Component* using DAVID (https://david.ncifcrf.gov/) and the list of all detected proteins (a) or the mouse genome (b) as background list. Asterisks indicate GO terms that contain extracellular proteins.

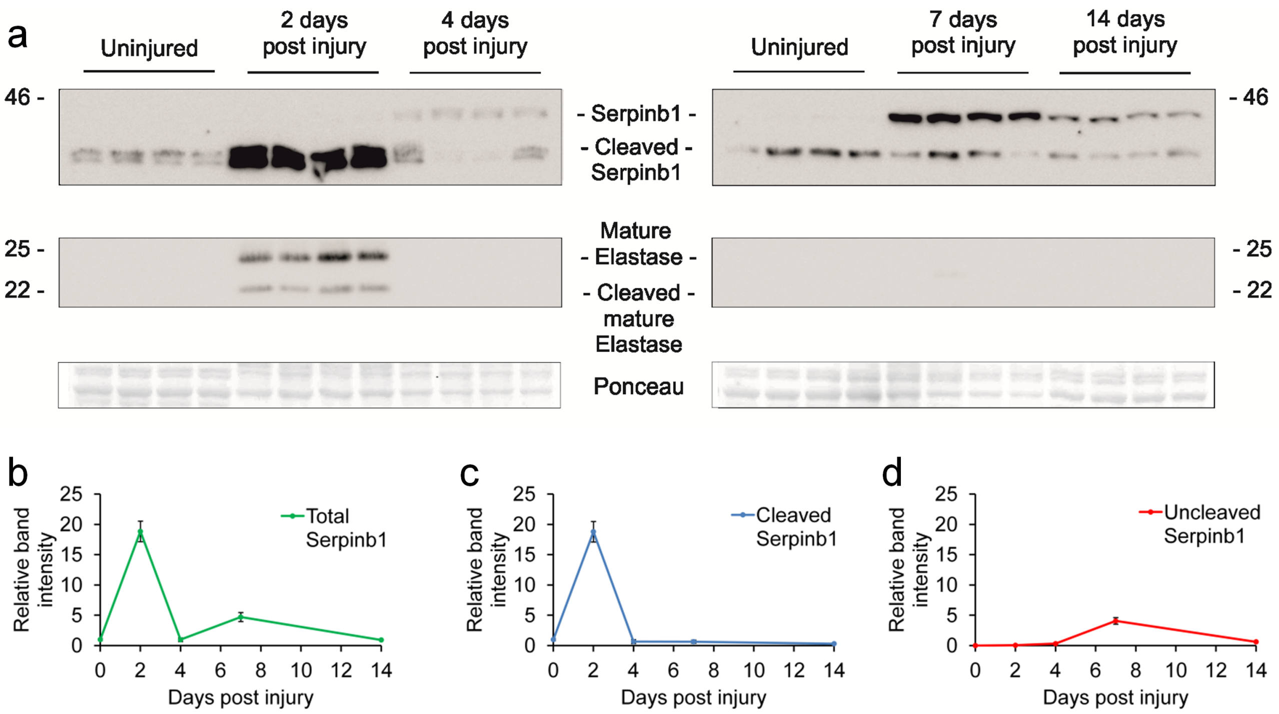

<u>Supplementary Figure S5</u>: Elastase and Serpinb1a levels are regulated during acute muscle injury. a) Western blot analysis of a muscle injury/regeneration time course. Due to the large number of samples, the four time points post-injury were split in two gels and the uninjured time point repeated on both gels for normalization. **b-d)** Quantification of (a) via densitometry image analysis (using ImageJ), specifically: (b) is a quantitation of the 42 kDa band (uncleaved Serpinb1a) + the 38 kDa band (cleaved Serpinb1a), (c) is a quantitation of only the 38 kDa band; (d) is a quantitation of only the 42 kDa band.

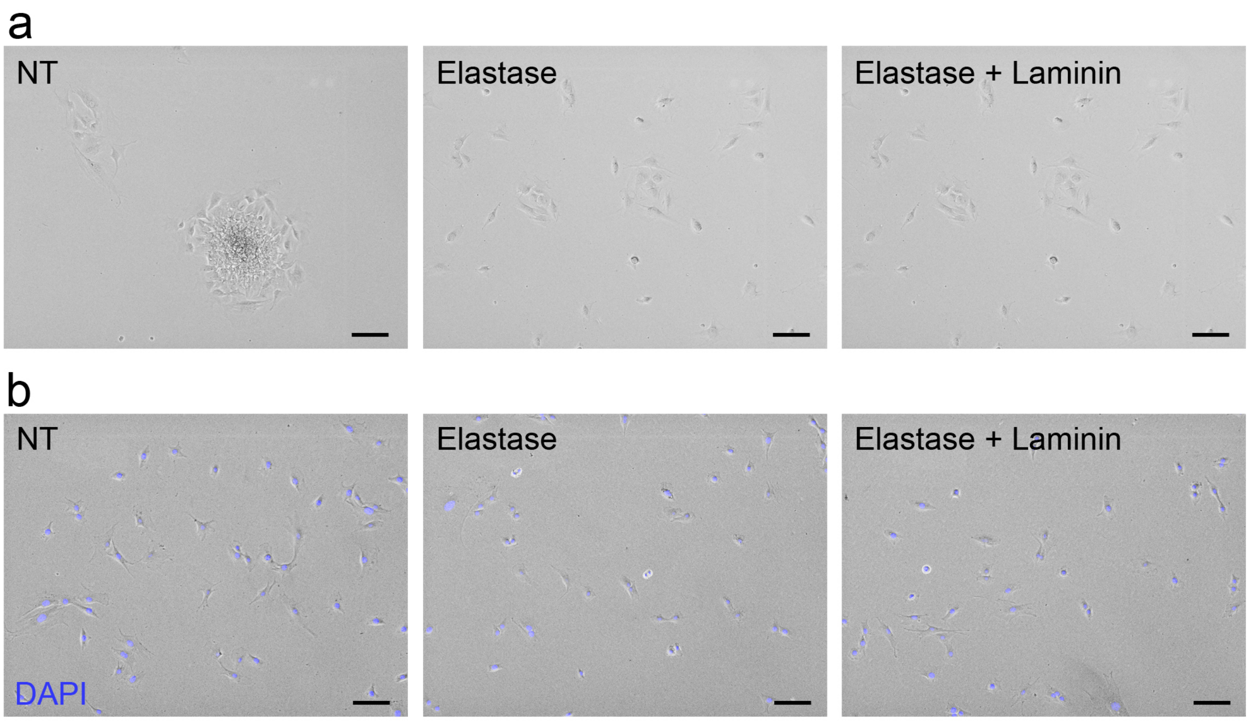

<u>Supplementary Figure S6</u>: **Elastase-degraded laminin is not toxic to myoblasts grown in suspension**. **a)** C2C12 myoblats were detached and resuspended in growth medium with/without the addition of elastase (0.6 U/mL) alone or elastase and laminin (50 µg/mL) as indicated. After 24 h culture in suspension in a humidified incubator (20% O_2, 5% CO_2) cells were plated on uncoated plates to test for cell viability. Non-treated cells (NT) had aggregated during the 24 hours in suspension while cells cultured in the presence of elastase (with or without laminin) had aggregated less. **b)** C2C12 myoblasts were cultured for 24 h as in (a) then centrifuged, resuspended in trypsin-EDTA 0.5% for 10 minutes at 37 °C, centrifuged again, resuspended in growth medium and plated to test for cell viability. No difference in cell numbers were observed between non treated cells and cells treated with elastase or elastase + laminin. Representative images from three independent experiments are shown. Scale bars are 100 µm.

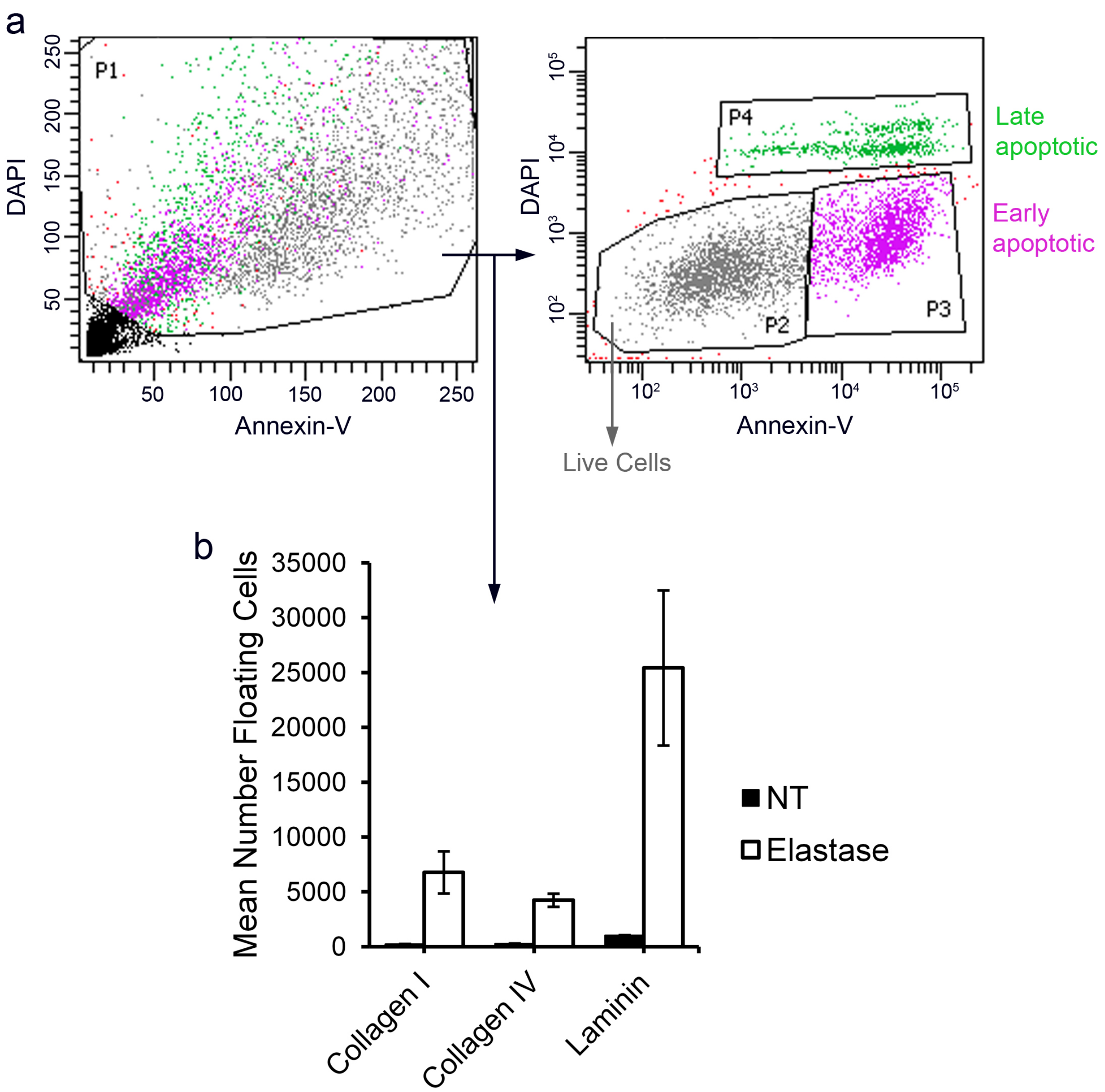

<u>Supplementary Figure S7</u>: **Flow Cytometry analysis of the elastase-induced detached cell population. a)** Gating scheme used to identify live, early apoptotic and late apoptotic cells via DAPI/Annexin-V staining. **b)** More cells had detached from adherent C2C12 cell cultures when treated with elastase compared to untreated. Despite the large effect size, p-values could not be accurately calculated due to the relatively small sample size (n=3 independent experiments, 1 measurement for each experiment thus, N=3).
